# Fine‐scale seascape genomics of an exploited marine species, the common cockle *Cerastoderma edule*, using a multimodelling approach

**DOI:** 10.1111/eva.12932

**Published:** 2020-02-24

**Authors:** Ilaria Coscia, Sophie B. Wilmes, Joseph E. Ironside, Alice Goward‐Brown, Enda O’Dea, Shelagh K. Malham, Allan D. McDevitt, Peter E. Robins

**Affiliations:** ^1^ Ecosystems and Environment Research Centre School of Science, Engineering and Environment University of Salford Salford UK; ^2^ School of Ocean Sciences Marine Centre Wales Bangor University Menai Bridge UK; ^3^ Institute of Biological, Environmental and Rural Sciences Aberystwyth University, Penglais Aberystwyth UK; ^4^ Met Office Exeter UK

**Keywords:** Irish Sea, larval dispersal, particle tracking, population connectivity, RADseq, redundancy analysis

## Abstract

Population dynamics of marine species that are sessile as adults are driven by oceanographic dispersal of larvae from spawning to nursery grounds. This is mediated by life‐history traits such as the timing and frequency of spawning, larval behaviour and duration, and settlement success. Here, we use 1725 single nucleotide polymorphisms (SNPs) to study the fine‐scale spatial genetic structure in the commercially important cockle species *Cerastoderma edule* and compare it to environmental variables and current‐mediated larval dispersal within a modelling framework. Hydrodynamic modelling employing the NEMO Atlantic Margin Model (AMM15) was used to simulate larval transport and estimate connectivity between populations during spawning months (April–September), factoring in larval duration and interannual variability of ocean currents. Results at neutral loci reveal the existence of three separate genetic clusters (mean *F*_ST_ = 0.021) within a relatively fine spatial scale in the north‐west Atlantic. Environmental association analysis indicates that oceanographic currents and geographic proximity explain over 20% of the variance observed at neutral loci, while genetic variance (71%) at outlier loci was explained by sea surface temperature extremes. These results fill an important knowledge gap in the management of a commercially important and overexploited species, bringing us closer to understanding the role of larval dispersal in connecting populations at a fine geographic scale.

## 1 INTRODUCTION

The assumption that genetic homogeneity predominates in marine organisms due to the lack of physical barriers and high dispersal potential at all life stages has been challenged in recent years (Allendorf, 2017). The advent of genomic markers generated by scanning the whole genome of an organism has equipped researchers with the necessary statistical power to detect more fine‐scale population differentiation within the marine realm (Benestan et al., 2015; Maroso et al., 2018). Nevertheless, there has been a shift in focus from neutral variation to adaptive genetic differentiation when studying marine organisms (Nielsen, Hemmer‐Hansen, Larsen, & Bekkevold, 2009). Markers under selection display higher divergent frequencies and can facilitate the identification of population structure and individual assignment (Araneda, Larraín, Hecht, & Narum, 2016; Nielsen et al., 2012; Woodings et al., 2018). They can also indicate the potential for resilience to environmental change (Razgour et al., 2019). Connectivity between marine populations is central to their health and resilience to external pressures such as parasites and pathogens (Rowley et al., 2014), pollution, human exploitation and climate change over ecological and evolutionary timescales (Burgess, Bowring, & Shen, 2014; Cowen & Sponaugle, 2009; Gimenez, 2019). For these reasons, it is vital to distinguish between neutral variation and adaptive divergence when attempting to understand what drives the observed population structure in marine organisms.

A seascape genomics approach is particularly valuable in this context (Grummer et al., 2019; Selkoe et al., 2016). Genetic and genomic data can be used in conjunction with environmental variables such as sea water temperature, salinity, water depth, irradiance, turbidity and sediment type (Viricel & Rosel, 2014) against neutral and adaptive genetic differentiation (Coscia, Robins, Porter, Malham, & Ironside, 2012; Young et al., 2015). This can provide new insights for interpreting genetic patchiness in relation to specific environmental features (Benestan et al., 2016; Bernatchez et al., 2019; Truelove et al., 2017).

Seascape genomics has proven to be particularly useful when considering exploited species, as it has the potential to inform management and aid sustainable exploitation by enabling management units to be defined (Bernatchez et al., 2017; Teacher, André, Jonsson, & Merilä, 2013). Despite intense exploitation of many marine species, their management rarely takes into account genomic information (Bernatchez et al., 2017; ICES, 2018) and exploited aquatic invertebrates (shellfish) in particular receive little attention from policymakers and stakeholders in comparison with fish (Elliott & Holden, 2017). In the Irish Sea, shellfish represent 80% of the total landings per year, with an economic value of £46.6 million (Elliott & Holden, 2017). Among these, the common cockle *Cerastoderma edule* fisheries are some of the most valuable fisheries for both Ireland and Britain (Dare, Bell, Walker, & Bannister, 2004; Hervas, Tully, Hickey, O’Keeffe, & Kelly, 2008) and are of high socio‐economic importance, valued at £3.3 million for Wales alone (Elliott & Holden, 2017).

The common cockle has both ecological and commercial importance, providing an important food source for wading birds in addition to employment for coastal communities (Flach & de Bruin, 1994; Hickin, 2013). *Cerastoderma edule* occurs in intertidal soft sediment regions of the eastern Atlantic, from Norway to Senegal. It can live for up to ten years and is characterized by high fecundity and high dispersal potential due to a pelagic larval phase which lasts for approximately 3–5 weeks following spawning from May to August (Malham, Hutchinson, & Longshaw, 2012). In recent decades, cockle stocks have declined across Europe, with production falling from 108,000 tons in 1987 to less than 25,000 tons in 2008 (Martínez, Mendez, Insua, Arias‐Pérez, & Freire, 2013). Cockle declines have been attributed to different factors in different locations, such as overharvesting (Wolff, 2005) and parasitic infections (Longshaw & Malham, 2015; Thieltges, 2006). In the UK, recurrent mass mortalities have occurred at several long‐established cockle fisheries, resulting in significant economic losses (Woolmer, 2013). These mortalities have not been attributed to any single environmental factor, and interactions between multiple stress factors are suspected (Callaway, Burdon, Deasey, Mazik, & Elliott, 2013; Malham et al., 2012). Sustainable management of the common cockle is hindered by poor understanding of their population connectivity. Analysis of microsatellite and mitochondrial DNA markers suggests weak barriers to gene flow between *C. edule* populations along the North European coast (Coscia et al., 2012; Martínez, Freire, Arias‐Pérez, Mendez, & Insua, 2015). However, these markers lack sufficient resolution to investigate connectivity at the finer scales relevant to fisheries management (Bernatchez et al., 2017).

Given the major logistical challenges of directly quantifying larval connectivity, efforts have focused on simulating ocean hydrodynamics to estimate larval dispersal (Cowen, Gawarkiewicz, Pineda, Thorrold, & Werner, 2007; Paris, Cowen, Claro, & Lindeman, 2005; Robins, Neill, Giménez, Jenkins, & Malham, 2013). This approach identifies well‐connected population groups as well as weakly connected, partially connected or isolated populations. These simulations highlight the importance of local and mesoscale hydrodynamics interacting with species‐specific larval behaviours in driving population persistence (Bode et al., 2019; Botsford et al., 2009; North et al., 2008; Robins et al., 2013) and recovery from stock decline (Gimenez, 2019). They highlight how the capacity of a population to recover from mass mortalities is contingent on the scale of disturbance relative to the scale of connectivity (Masier & Bonte, 2019). Usual circulation patterns, and hence connectivity, can be modulated by severe wind and wave conditions. Previous larval dispersal studies have predicted that given atypical meteorological conditions during spawning events, new connectivity routes can be established (e.g. by reversing the Celtic Sea front circulation (Hartnett, Berry, Tully, & Dabrowski, 2007) or by large‐distance displacements from expected routes affecting sea turtles (Monzón‐Argüello et al., 2012)).

In the present study, a seascape genomics approach using single nucleotide polymorphisms (SNPs) was employed, for the first time, to resolve patterns of population structure of the common cockle between estuaries within a commercially active area (the Irish and Celtic seas), with the goal of identifying management units. For this purpose, we first tested for neutral population structure and then assessed the role of current‐mediated larval dispersal in shaping it. We then investigated the relationship between environmental factors and adaptive divergence, after identifying outlier markers, with a particular focus on abiotic factors such as water temperatures (surface) and ocean stratification during the spawning season. To do this, larval transport between sites was estimated and connectivity matrices derived from oceanographic modelling, accounting for interannual variability due to biophysical parameters, like spawning time and larval duration.

## 2 MATERIALS AND METHODS

### 2.1 Sampling and DNA extraction

**Figure 1:**
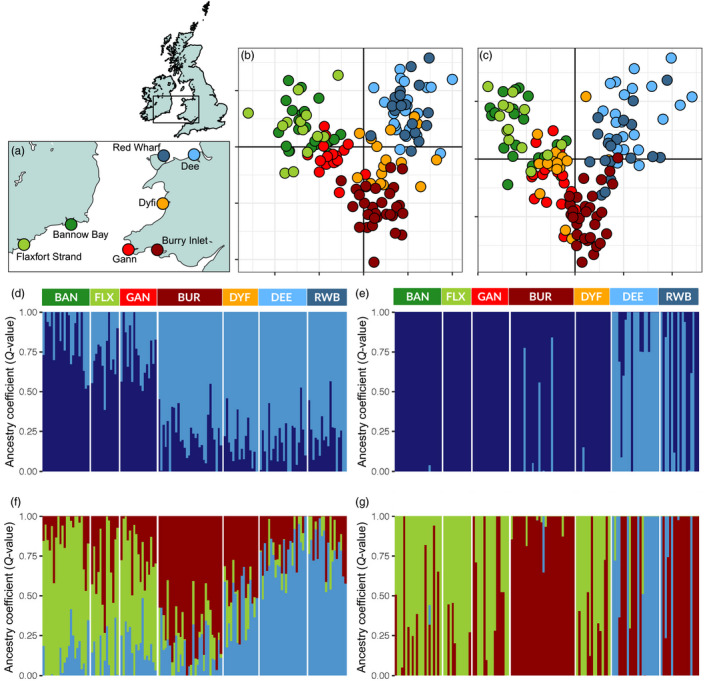
(a) Sampling locations; (b) DAPC using the neutral data set; (c) DAPC using the 14 outliers; below, the barplots generated by sNMF from the neutral (d, f) and outlier (e, g) data sets, for both *K* = 2 and *K* = 3

Cockles were collected during 2010 and 2011 from seven locations off the coasts of Ireland (Bannow Bay [BAN] and Flaxfort Strand [FLX]) and Britain (Burry Inlet [BUR], Gann Estuary [GAN], Dyfi Estuary [DYF], Red Wharf Bay [RWB] and Dee Estuary [DEE]) (Figure 1 and Table 1). Genomic DNA was extracted from ethanol‐preserved and frozen tissue using the DNeasy Blood and Tissue Kit with an additional RNase A step (Qiagen©). The quality and quantity of the extracted DNA were assessed by gel electrophoresis (1% agarose) and Qubit dsDNA HS (high sensitivity, 0.2–100 ng) Assay Kit on a Qubit 3.0 fluorometer (©Thermo Fisher).

**Table 1:** Genetic diversity indices for the two data sets.

		*N*	Neutral			Outlier		
			*H*_O_	*H*_E_	*F*_IS_	*H*_O_	*H*_E_	*F*_IS_
Bannow Bay	BAN	22	0.133	0.150	**0.087**	0.269	0.272	0.006
Gann	GAN	17	0.129	0.147	**0.094**	0.246	0.240	0.032
Dee	DEE	22	0.116	0.148	**0.173**	0.218	0.355	**0.335**
Dyfi	DYF	11	0.103	0.149	**0.238**	0.146	0.234	**0.394**
Flaxfort Strand	FLX	13	0.103	0.156	**0.275**	0.197	0.266	**0.201**
Red Wharf	RWB	18	0.118	0.144	**0.141**	0.286	0.337	**0.179**
Burry Inlet	BUR	30	0.109	0.150	**0.230**	0.173	0.218	0.159

Abbreviations: *F*_IS_, the inbreeding coefficient; *H*_E_, expected heterozygosity; *H*_O_, observed heterozygosity; *N*, number of individuals remaining after filtering for each location.

Values that are significant (95% confidence interval) are in bold.

### 2.2 RAD sequencing and bioinformatic analysis

Reduced representation libraries (Baird et al., 2008) were constructed using the restriction enzyme *PstI* (New England Biolabs) for restriction site‐associated DNA sequencing (RADseq). RAD libraries (each consisting of 24 uniquely barcoded individuals) were produced according to Etter, Preston, Bassham, Cresko, and Johnson (2011). Each library was quantified using real‐time PCR and single‐end (100‐bp target)‐sequenced in‐house on an *Illumina HiSeq 2000*.

Initial bioinformatic analysis, including quality control, demultiplexing and identification of polymorphisms, was performed by *Floragenex* (www.floragenex.com), using *Samtools* 0.2 (Li et al., 2009) and custom scripts, retaining one SNP per tag, with a minimum coverage depth of six for each allele, and genotyped in at least 70% of the individuals in the overall data set. These settings produced an initial data set of 191 individuals and 4,271 single nucleotide polymorphisms (SNPs), which was then further filtered by the authors using the packages *poppr 2.8.1* (Kamvar, Brooks, & Grünwald, 2015; Kamvar, Tabima, & Grünwald, 2014) and *adegenet* (Jombart, 2008; Jombart & Ahmed, 2011) in *R* (R Core Team, 2019). Markers with data missing in more than 25% of individuals were discarded from the data set, as well as loci with *F*_IS_ equal to 1, −1 or NA. Three MAF (minimum allele frequency) filters of 0.05, 0.025 and 0.01 were then applied, generating three data sets. No significant effect of MAF filter upon heterozygosity, global *F*_ST_ and population structure was detected, and so a MAF filter of 0.01 across all sites was applied (Xuereb et al.., 2018). This allowed us to maximize the number of markers and hence the information available at fine spatial scale, while reducing the bias that might be introduced by retaining low‐frequency SNPs (Roesti, Salzburger, & Berner, 2012). All the downstream analyses were thus performed on the MAF = 0.01 data set. Markers in linkage disequilibrium (LD) were identified and removed using the function *pair.ia* of the *poppr* package (*r* ^2^ > .7). Finally, SNPs that were found to deviate from Hardy–Weinberg equilibrium (HWE; *p = *.01) in four out of seven populations were removed (Wyngaarden et al., 2018). The final, filtered data set included 138 individuals from the seven locations, and 1,725 SNPs (Table S1).

### 2.3 Neutrality tests and population structure

Genomic markers were tested for neutrality using two complementary methods: *BayeScan* 2.1 (Foll & Gaggiotti, 2008) and the R package *pcadapt* (Luu, Bazin, & Blum, 2017)*.* In order to minimize the detection of false positives, we considered as outliers only those SNPs that were selected by both methods (Coscia et al., 2012). *BayeScan* is an *F*_ST_‐based, Bayesian method (Beaumont & Balding, 2004), while *pcadapt* is based on a principal component analysis (PCA) of individual genotypes and is known to perform particularly well in the presence of weak structure, admixture or range expansions (Luu et al. 2017). *BayeScan* was run using default settings (5,000 iterations, 10 thinning intervals, 20 pilot runs—5,000 iterations each, 10 prior odds). For *pcadapt*, we chose the most appropriate number of clusters K in the scree plot, which displays in decreasing order the percentage of variance explained by each principal component (PC). *Q*‐values were used to account for false discovery rate, and SNPs were considered as significant outliers at alpha values ≤.05. Genetic diversity was estimated on three data sets: overall, neutral and outliers, in order to disentangle the role of demographic processes versus selection. Heterozygosity (expected and observed) was estimated with the R package *hierfstat* (Goudet, 2005; Goudet & Jombart, 2015), while population pairwise *F*_ST_ (Weir & Cockerham, 1984) and relative 95% confidence interval (1,000 bootstraps) were estimated with the R package *assigner* (Gosselin, 2019).

Individual‐based population structure was assessed using two approaches: discriminant analysis of principal components (DAPC) as implemented in *adegenet* (Jombart, 2008; Jombart & Ahmed, 2011) and the function *sNMF* of the LEA package (Frichot & Francois, 2015). While DAPC uses a priori spatial information (sampling locations), sNMF does not make any assumptions about sampled and ancestral populations. The number of K clusters in the data sets was chosen using the Bayesian information criterion (Schwarz, 1978) in DAPC. In sNMF, the entropy criterion provided a basis for choosing the number of ancestral populations that best explain the genotypic data (Alexander & Lange, 2011; Frichot, Mathieu, Trouillon, Bouchard, & Francois, 2014).

The power of the neutral and outlier data sets to discriminate and assign individuals was determined with a genotype accumulation curve (Figure S2), calculated with the function *genotype_curve* of the *poppr* package.

### 2.4 Hydrodynamic modelling

**Figure 2:**
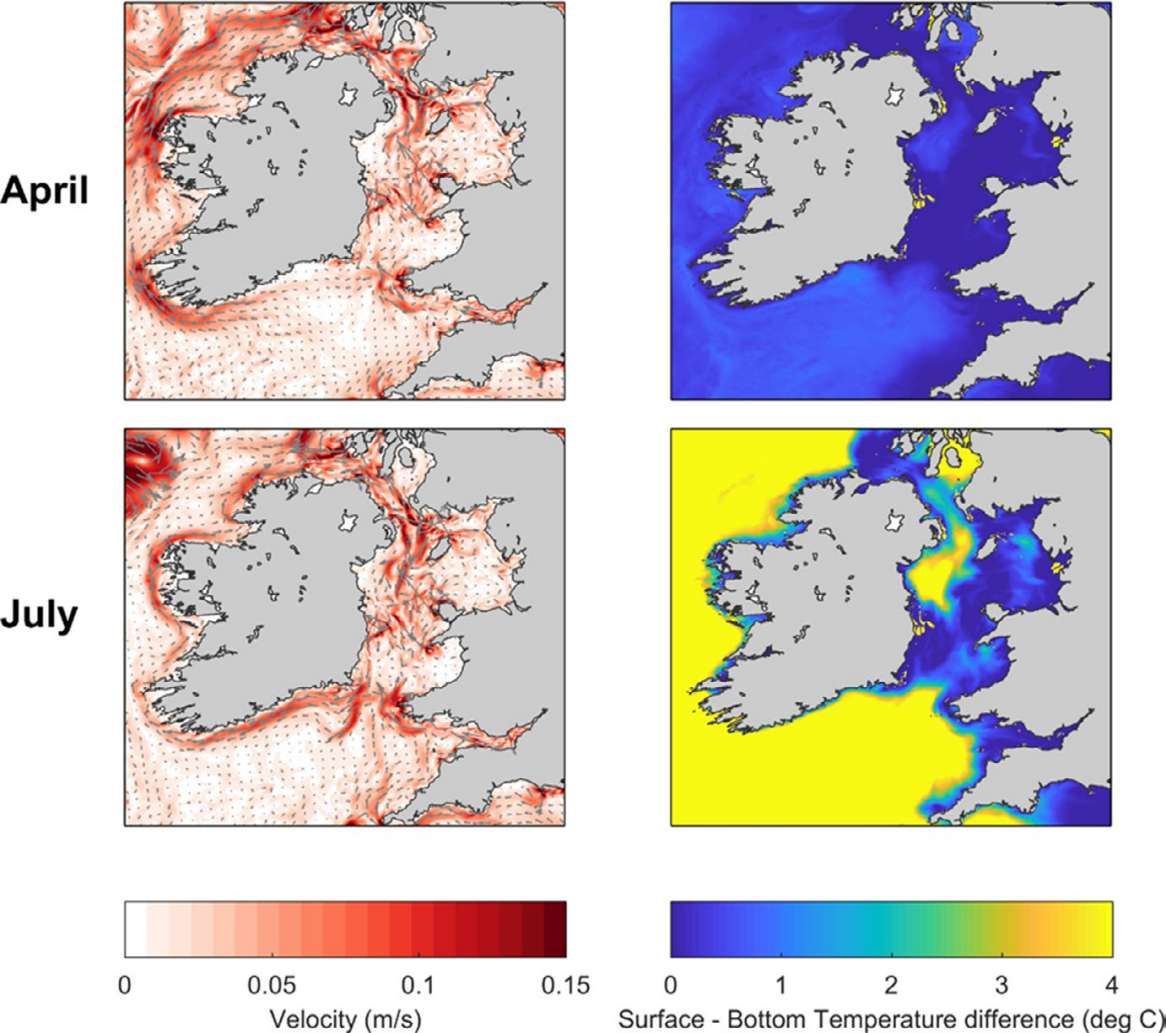
(Left) Simulated mean depth averaged ocean current strength in the Irish and Celtic seas for April (top) and July (bottom) 2014. The magnitude of the currents is shaded in red, and the direction of the currents is indicated with the grey arrows. (Right) Simulated mean surface to bottom temperature difference for April (top) and July (bottom) 2014. This is a measure of the degree of stratification of the water column with blue colours resembling a more mixed water column and yellows a more stratified water column

Simulations of 3D flow fields were used to drive a particle tracking model (PTM) developed to simulate potential larval transport and connectivity between the seven sampling locations. The modelling considered natural variability in larval dispersal caused by the timing of spawning relative to the lunar tidal cycle and the variability of ocean currents over seasonal and interannual timescales. Each PTM simulation time was varied from 20 to 40 days to reflect the pelagic larval duration of *C. edule* (Malham et al., 2012). Simulated flow fields in the Irish and Celtic seas were obtained from the NEMO Atlantic Margin Model (AMM15). The model was developed to resolve key dynamic features of the European north‐west shelf including the influence of shelf‐break dynamics. It has a horizontal resolution of 1.5 km and 51 terrain‐following vertical layers. Simulated velocities accounted for interannual variability (Grummer et al., 2019) and were obtained for the years 2008–2014, at daily‐averaged resolution (Figure S7). For the year 2014, simulated velocities were output during the cockle spawning/settlement season (April–September) at hourly averaged temporal resolution. While our results focus on the higher resolution data (2014 hourly averaged), we compare our results with the interannual daily‐averaged data (Figure 2). Further details of the model set‐up and validation are presented in the Supplementary Material.

### 2.5 Particle tracking model (PTM)

The particle tracking model simulates the lagrangian movement of individual particles in space and time, based on oceanographic dispersion and individual particle behaviour. In our case, particle behaviour was represented by varying the pelagic larval duration to reflect the observations in the field (Malham et al., 2012). However, given the paucity of data about *C. edule* larval behaviour, the effects of vertical swimming were not incorporated into the PTM. The PTM was programmed in Python.

**Figure 3:**
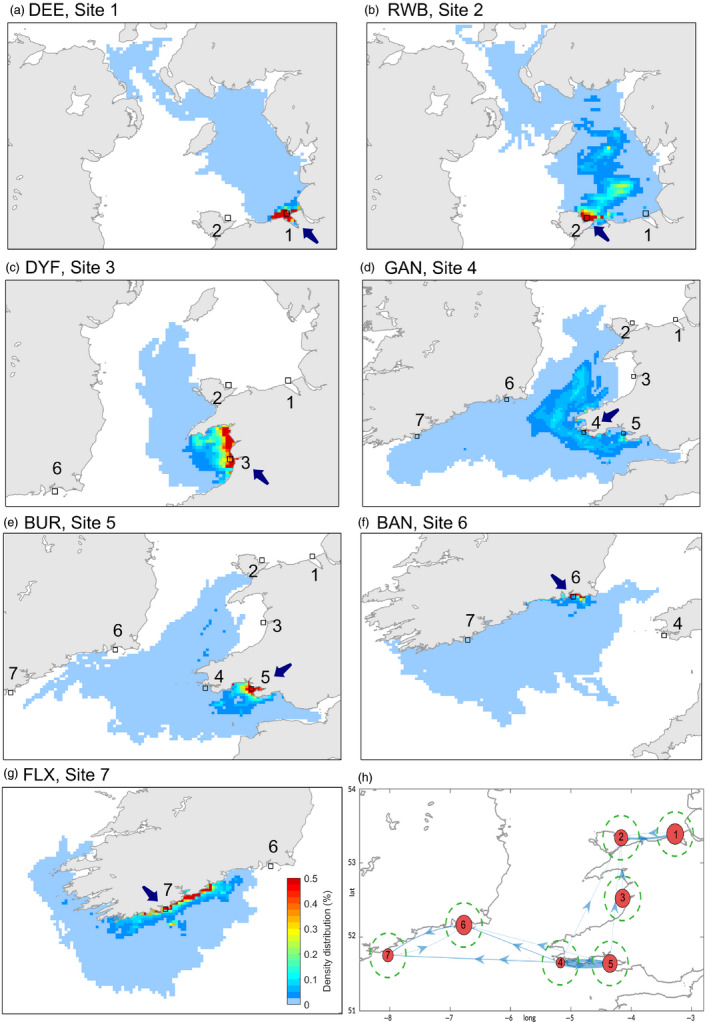
(a–g) Probability density distribution maps for 2014 showing simulated dispersal probability from release Sites 1–7 (black squares). Each panel shows dispersal probability for 11,520,000 particles (12,000 each month × 6 months × 10 settlement days). (h) Seasonally‐averaged connectivity networks: the thickness of the pathways in corresponds to the average modelled connectivity. The size of the red circles corresponds to self‐recruitment, and the dashed green circle show the settlement radius used to estimate connectivity

A previous sensitivity study by Robins et al. (2013) demonstrated that cohorts of 10,000 particles released from a range of locations within the Irish Sea were sufficient to statistically represent dispersal and connectivity patterns. Accordingly, cohorts of 750 particles were released from Sites 1–7 (Figure 3), each day at 12:00, over the first 16 days of April 2014, that is a total of 12,000 particles per site, with releases spanning a spring–neap tidal cycle. These simulations were repeated from April to September 2014. Due to interannual variations in weather patterns, the timing of the onset of seasonal stratification and its strength can vary between years. To assess this, the procedure was repeated for 2008–2014 using daily‐averaged velocity fields. The dispersal of each particle was tracked for 40 days’ pelagic larval duration (PLD). Particles were neutrally buoyant and able to disperse throughout the 3D flow field. Connectivity between populations was determined from particle trajectories during days 30–40; particles that travelled within 10 km of a settlement site were assumed to have settled there. The 10 km threshold was based on the average tidal excursion for the Irish Sea (Robins et al., 2013), although a range of thresholds are discussed in the Section 3.

### 2.6 Spatial eigenfunction analysis (SEA) and environmental association (EA) analysis

To test for association between the oceanographic environment and the cockle genetic structure, a spatial eigenfunction analysis (SEA) (Dray et al., 2012) was performed. This approach was implemented through the *vegan* and *adespatial* packages (Dray et al., 2019; Oksanen et al., 2017) in R. This method allowed us to estimate the influence of geographic distance between samples as well as the influence of modelled connectivity on genomic variation.

Geographic distance was represented as distance‐based Moran's eigenvector maps or *dbMEM* (Stéphane Dray, Legendre, & Peres‐Neto, 2006). dbMEMs were calculated using the *PCNM* function of the *vegan* package on the Euclidean distances (calculated with the function *dist*) in turn estimated from Cartesian‐transformed coordinates using the *geoXY* function in the *SoDA* package (Chambers, 2013). *PCNM* transforms spatial distances in rectangular matrices that are suitable for constrained ordination (Legendre & Gallagher, 2001).

For the environmental association analysis (EA), simulated connectivity was represented as asymmetric eigenvector maps or AEM (Blanchet, Legendre, & Borcard, 2008; Blanchet, Legendre, Maranger, Monti, & Pepin, 2011). AEM is a spatial eigenfunction approach specifically developed to model multivariate responses to asymmetric and directional processes such as current‐driven larval dispersal (Blanchet et al., 2011). The connectivity probability matrix generated by the particle tracking model was translated into a nodes‐to‐edge matrix which records the presence/absence of connectivity links (through *edges*, 19 here) between *nodes* (the 7 sampling locations). Each edge has an associated “weight,” based on the simulated probability of connectivity. AEMs were calculated with the *aem* function in R.

For each of the seven sampling locations, the simulated monthly averaged sea surface temperature (SST) and surface–bottom temperature difference (SBTD—the difference between the temperature at the surface and the bottom of the water column) representing ocean stratification, were extracted from the ocean model (Figure S1). The relative contribution of temperature (SST and SBTD), geographic distance (dbMEMs) and simulated larval connectivity (AEMs) to genetic variation in both the neutral and the outlier data sets (response variable) was estimated using redundancy analysis (RDA). In particular, the response variable was represented by population‐specific minor allele frequencies (MAF) of each SNP calculated in the R package *hierfstat* (function *minorAllele*) (Goudet & Jombart, 2015), detrended using the *decostand* function with the *hellinger* transformation available in *vegan* (Oksanen et al.,^2019^). The most important explanatory variables were chosen by performing the forward selection with 10,000 permutations in *vegan* using the function *ordistep.* The significance of the results was assessed with an analysis of variance (function *ANOVA* in *vegan*) with 1,000 permutations, to finally establish which factors were most correlated with genetic variation. Redundancy analysis (RDA) and partial RDA (corrected for geographic distance between populations) were performed on the putatively neutral (1711) and outlier (14) SNP data sets, and parsimonious RDAs were carried out using the variables selected (Borcard, Gillet, & Legendre, 2011).

## 3 RESULTS

### 3.1 Genetic diversity and population structure

After filtering for missing data, *F*_IS_, minimum allele frequency, Hardy–Weinberg equilibrium and linkage disequilibrium, 138 individuals and 1725 SNPs were retained (Table S1). *BayeScan* detected 28 SNPs as potential outliers, with a False Discovery Rate of 0.05, whereas *pcadapt* found 62. Only 14 SNPs overlapped between the two approaches. The downstream analyses were then carried out on two data sets: neutral (1711 SNPs) and outliers (14 SNPs).

For the neutral data set, expected and observed heterozygosity (*H*_E_ and *H*_O_) were similar across locations, ranging between 0.148–0.157 and 0.103–0.134, respectively (Table 1). Pairwise *F*_ST_ (Figures S3 and S4) ranged between 0 (Red Wharf Bay–Dee) and 0.0289 (Gann–Dee). *sNMF* detected three clusters using the entropy criterion (*k* = 3; Figure 1d‐G), while *DAPC*’s *find.clust* found a maximum of five clusters (*k* = 5; Figure 1b,c) based on the BIC scores. For the putative outlier data set, *F*_ST_ values ranged between 0 (Dyfi–Gann) and 0.38 (Burry–Flaxfort Strand), but *DAPC*’s *find.clust* indicated that one cluster explained the structure in the data. All population‐level neutral *F*_IS_ values were significant (95% confidence interval) and positive, ranging between 0.08 and 0.27 (Table 1). For the outlier data set, *H*_E_ and *H*_O_ varied respectively between 0.21–0.35 and 0.14–0.28 and *F*_IS_ values were positive and significant for four populations: Dee, Red Wharf Bay, Dyfi and Flaxfort Strand.

Genotype accumulation curves (Figure S2) for both data sets—neutral and outlier—show that a plateau is reached and variance decreased, indicating that the data sets have sufficient power to distinguish between individuals (Arnaud‐Haond, Duarte, Alberto, & Serrão, 2007).

### 3.2 Seasonal circulation and larval dispersal

The simulated circulation structure within the Celtic and Irish seas has, in our study, contributed markedly to distinct patterns of larval transport and population connectivity that would not be anticipated from geographic location alone. Moreover, spatial variability in these patterns was simulated over seasonal timescales. The strong tides that characterize this region and have the potential to advect larvae tens of kilometres away but are oscillatory, resulting in minimal net larval dispersal (Robins et al., 2013; Robins et al. 2015). Thus, larval transport is controlled by density‐driven currents (or lack of) along frontal boundaries that develop due to thermal stratification during summer months (Horsburgh, Hill, & Brown, 1998; Simpson & Hunter, 1974). Although much weaker than tidal currents, these density‐driven currents are persistent over time and act as key pathways for dispersing larvae. For example, the Celtic Sea front may facilitate connectivity of cockle populations from South Wales across to Ireland (Coscia et al., 2012), but also restrict transport across the front between the Celtic and Irish seas.

Since our cockle samples were collected during 2010–2011, we ensured that 2014 was representative of the oceanographic conditions by carrying out PTM simulations using daily current data from 2008 to 2014. Correlations between dispersal maps for the 2014 hourly versus daily current data were high (*r* ^2^ > .87) for the majority of sites. The largest differences were found for Red Wharf Bay, where particles dispersed similar distances but north‐eastwards in the hourly runs and north‐westwards in the daily simulations; and Flaxfort Strand, where differences in the degree of retention close to the Irish coast can be seen. However, for all sites, correlations between connectivity networks for the 2014 hourly versus daily current data were high (*r* ^2^ > .95). For the daily current data spanning 2008–2014, dispersal maps for all sites and years (Figures S7.1–S7.7) were correlated by a minimum of *r* ^2^ > .83 (mean *r* ^2^ = .97) (Figure S8), suggesting very little interannual variability. A similar pattern was found for the yearly (2008–2014) connectivity matrices which were correlated with each other year on average with an *r* ^2^ of .99.

The AMM15 simulation showed two distinct circulation patterns. Firstly, in April, the region is vertically well mixed and mostly governed by tides (Pingree & Griffiths, 1979) (Figure 2). These net flows were generally weak (<0.1 m/s) and directed west around southern Ireland and north in the Irish Sea. Secondly, from May to September, density‐driven currents generated by the inset stratification started to develop (Figure 2 shows July). Persistent east‐to‐west currents developed along the Celtic Sea front and an anticlockwise gyre formed in the western Irish Sea. Along these fronts/gyres, the strongest flows were ~0.2 m/s at the thermocline at ~30 m depth.

The degree of exposure of the spawning grounds (e.g. open coast or enclosed bay) influenced the population's ability to connect with other sites (Figure 3 and Figure S5). This is shown by comparing the simulated particle dispersal from Dee (Site 1, enclosed bay) with Red Wharf Bay (Site 2, open coast) on the north Wales coast. The majority of particles dispersing from DEE remained <30 km from the release site, resulting in high proportions of self‐recruitment (17.4 ± 0.7%), with small proportions of particles dispersed further to the Scottish coast (Figure 3a and Figure S5). In contrast, particles from Red Wharf Bay were exposed to stronger currents and the site experienced lower self‐recruitment (13.6 ± 3.5%) with “hot spots” of higher density particles (up to 0.5%) advected ~100 km northwards (Figure 3b).

Our simulations suggest a high degree of isolation at Dyfi (Site 3; Figure 3c and Figure S5), with the majority of particles retained in close proximity. At the same time, due to the weak flows, simulated particles from other sites never reached this site. Particles from Gann (Site 4) and Burry (Site 5) were capable of dispersing readily between one another (up to 1% connectivity) and also westwards to the Irish populations, particularly from the more exposed Gann Estuary (up to 0.5% connectivity) (Figure 3d,e and Figure S5). Connectivity with Dee was possible but unlikely (<0.01%). It was clear from the simulations that seasonal patterns of dispersal are expected, for example from Bannow Bay (Site 6) and Flaxfort Strand (Site 7) (Figures S5 and S6). Simulated dispersal from these populations was caught in the persistent westward currents around southern Ireland, with the particles generally travelling further west as the residuals strengthened during summer months. For Bannow Bay, only during April when the Celtic front had not formed, were particles able to disperse northwards from the Celtic into the Irish Sea.

### 3.3 Environmental association analysis

The *ordistep* function found that three variables were linked to the population structure found at neutral loci: (a) July SBTD explained 10% of the genetic variance (*p* = .002, adj*R* ^2^ = .10); (b) geographic distance (dbMEM‐1) explained 7% (*p* = .000, adj*R* ^2^ = .07); and (c) simulated connectivity (AEM2) explained 4% (*p* = .050, adj*R* ^2^ = .04). Partial RDAs on neutral data using these three variables were not significant.

**Figure 4:**
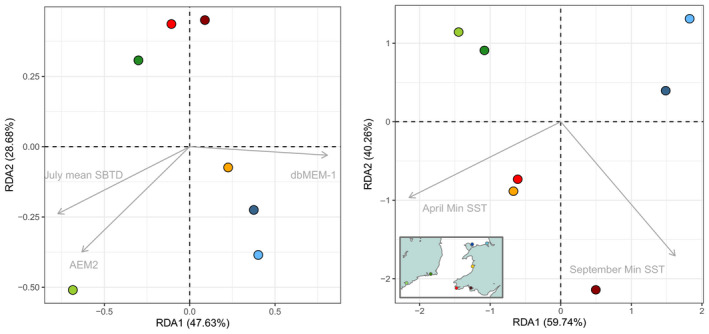
Redundancy analysis (parsimonious RDA) performed for the (a) neutral and (b) outlier data sets. Each circle is a sampling location, and each arrow is an environmental variable that significantly drives the observed population structure

For the outlier data set, two environmental factors were found to be significant, explaining 71% of the genetic variance: April min SST (*p* = .025, adj*R* ^2^ = .71) and September min SST (*p* = .004, adj*R* ^2^ = .71). These SST values were the lowest daily mean SST in each month (Figure S1). The global parsimonious RDA (Figure 4a) was overall nonsignificant when including the three selected factors at once (*p* = .11), although it was significant when including two at a time: geographic distance (dbMEM‐1) + July mean SBTD (*p* = .008), and simulated connectivity (AEM2) + geographic distance (dbMEM‐1) (*p* = .022). Partial RDAs were not significant. The parsimonious RDA run on outliers was globally highly significant (*p* = .002; Figure 4b).

For the neutral RDA, the first axis explained almost 48% of the total variance and was mainly driven by geographic distance (dbMEM‐1) between sites. This axis separates the northern sites of Red Wharf Bay, Dee Estuary and Dyfi Estuary from the southern sites of Flaxfort Strand (FLX), Bannow Bay (BAN), Gann Estuary (GAN) and Burry Inlet (BUR), describing a north–south divide that is not connected by oceanic currents. The second axis (RDA2) was influenced predominantly by an asymmetrical eigenvector representing simulated connectivity (AEM2) and July mean SBTD, a proxy for maximum seasonal ocean stratification. On this axis, the southernmost population Flaxfort Strand (light green) is strongly related to simulated larval connectivity (AEM2) (Figure 4a). Redundancy analysis carried out on the outlier data set revealed patterns of adaptive divergence, with cooler sea surface temperatures being the most significant driver (Figure 4b).

## 4 DISCUSSION

This is the first study to use genomic markers and biophysical larval transport simulations to investigate the drivers of connectivity in a commercially important shellfish in north‐western Europe. This research was conducted in the Irish and Celtic seas where *C. edule* forms valuable shellfisheries for both Ireland and Britain. A panel of 1,725 SNP markers were analysed in relation to sea temperature and oceanographic currents, environmental variables that have been shown to be drivers of population structure in bivalves (Araneda et al., 2016; Bernatchez et al., 2019; Gormley et al., 2015; Lehnert et al., 2019; Xuereb et al., 2018). Using neutral genomic markers, three main genetic groups were identified, which can be considered stocks or management units. The first group includes the north Wales populations of Red Wharf Bay and Dee Estuary, the second includes the Irish Bannow Bay and Flaxfort Strand and the Welsh Gann Estuary, and the final group contains only the Burry Inlet on the south coast of Wales. To our knowledge, this is the first time such a pattern has been detected in *C. edule*, or in any other shellfish species in the Irish Sea.

Overall, the genetic results fit well with the predictions of the larval transport model, providing a level of empirical validation for both the simulated hydrodynamics and connectivity by larval dispersal. Environmental association analysis revealed that neutral genetic structure was strongly linked to geographic distance between sites and to the strength and direction of the ocean currents acting as corridors for larval dispersal, whereas colder periods (cold SSTs) were identified as potential drivers of adaptive divergence.

### 4.1 Connectivity, fine‐scale population structure and adaptive divergence

Neutral genetic diversity was low across the cockle populations within the study area, and low compared with previous studies on marine bivalve population genomics (e.g. Bernatchez et al., 2019; Lehnert et al., 2019). Populations of cockles in the Irish Sea have been under pressure for at least two decades, with mass mortality events and declines due to overexploitation leading to strict management of most beds across the UK (Woolmer, 2013). In addition, variance in reproductive success is known to occur in bivalves (Hedgecock & Pudovkin, 2011). These events could be responsible for the loss of genetic diversity, as already observed in several marine organisms (Pinsky & Palumbi, 2014). On the other hand, heterozygote deficiency (as indicated by positive *F*_IS_ in all sampling locations; Table 1) is well known to occur in marine bivalves (Gaffney, 1994), and had already been detected in these cockle populations using microsatellites (Coscia et al., 2012). Furthermore, cockles have been shown to undergo boom and bust years (Morgan, O’Riordan, & Culloty, 2013) with the dispersal of cockle larvae and recruitment altering between years (Miller, Versace, Matthews, Montgomery, & Bowie, 2013; Morgan et al., 2013). Another explanation of positive *F*_IS_ could be the Wahlund effect, caused by the presence of substructure within populations. This was not considered to be the case in this data set, given that no subpopulation pattern was found in the clustering analysis (Manangwa et al., 2019).

Given the fine spatial scale and the reproductive biology of the study organism (broadcast spawner with a long pelagic larval phase), a lack of or weak population structure was expected. Previous studies in the same geographic area identified a lack of genetic structure in shellfish species using microsatellite markers (Coscia et al., 2012; McKeown, Watson, Coscia, Wootton, & Ironside, 2019; Watson, McKeown, Coscia, Wootton, & Ironside, 2016).

Geographic proximity certainly favours gene flow, but oceanographic currents aiding larval transport are also major drivers of population structure (Barbut et al., 2019). For example, Red Wharf Bay and the Dee Estuary appear to be genetically homogeneous, due to high levels of gene flow, but these populations are very distinct from those further south (150–350 km away). This concurs with the model's prediction that larvae from the north coast of Wales will disperse northwards. The high levels of gene flow detected between the Gann Estuary on Wales’ south‐west coast and sites on the south‐east coast of Ireland also corresponds with the model's prediction of high levels of westward dispersal along the Celtic Sea front (Coscia et al., 2012).

Of particular interest is the genetic make‐up of the population in the Burry Inlet. Here, cockles have experienced recurrent mass mortality events for 15 years (https://marinescience.blog.gov.uk/2015/08/14/unusual-cockle-mortalities-burry-inlet/). The relatively high level of genetic differentiation between the neighbouring Burry Inlet and Gann Estuary populations indicates that gene flow between these populations is low, seemingly contradicting the larval transport model's prediction of high connectivity. This result suggests that the Burry Inlet population may able to persist through self‐recruitment, rather than forming a sink population depending upon immigration from healthier populations elsewhere, and could explain the low genetic diversity and high levels of inbreeding detected there. However, the model showed that larval dispersal is possible from the Gann to Burry estuaries, although we assumed a relatively large settlement zone that extended beyond the mouth of the Burry Inlet (Figure S9). Furthermore, it must be acknowledged that the Gann Estuary cockle population is far smaller than that of the Burry Inlet so larvae dispersing from the Gann into the Burry may be swamped by self‐recruitment or might not survive due to local selection against them (i.e. density dependence; Ford, Shima, & Swearer, 2016). Additionally, the southern coast of Wales contains other large cockle populations, such as the Three Rivers fishery midway between the Gann and Burry. These populations were not sampled/modelled in this study but may provide a nearer and greater source of larvae for the Burry Inlet than does the Gann Estuary.

The spatial environmental association analysis identified several environmental factors that appeared associated with the population structure observed at neutral and outlier genetic markers. This is a strong statistical approach, which has already been successfully employed to study the influence of the environment on the genetic structure of commercially important bivalves, such as the eastern oyster (*Crassostrea virginica*; Bernatchez et al., 2019) and Atlantic deep‐sea scallop (*Placopecten magellanicus*; Lehnert et al., 2019). In *C. edule,* neutral genetic structure is strongly dependent on geographic distance between sites (dbMEM‐1), indicating that isolation by distance plays an important role in shaping the observed genetic structure in this species, despite its long pelagic larval duration. Nevertheless, it is the interplay between isolation by distance, extremes in temperature‐driven currents (July SBTD) and simulated connectivity (AEM2) that shapes neutral population structure. The summer stratification which strengthens the Celtic Sea front current, directed from south Wales to Ireland (Simpson & Hunter, 1974), plays a major role in connecting cockle populations between the south of Wales and Ireland, while separating them from populations further north in the Irish Sea. Given the results of the larval dispersal modelling, the lack of significant associations between most simulated AEMs and the genetic structure revealed by the RDA was surprising. The weak performance of the model in predicting the observed genetic structure may be because it simulates direct connectivity between populations over one generation, while in natural populations connectivity and gene flow are built over multiple years (generations), often via intermediary populations (in a stepping stone manner).

Furthermore, our connectivity matrices (Figure 3 and Figure S5) were not able to take into account the population abundance of cockles and hence the number of larvae produces at each site.

Our modelling results suggest that larval dispersal is stable on interannual timescales (2008–2014). However, a degree of interannual variability was detected along exposed coasts and in regions of fronts (e.g. Red Wharf Bay and Flaxfort Strand). Although this variability does not affect our results because the populations were coastal and generally, a high level of coastal retention can be seen for most sites, other species that spawn offshore or have longer PLDs (e.g. scallops or lobsters) would likely show higher interannual variability in dispersal and connectivity and hence population structure. This would be expected especially of populations close to oceanographic fronts that are affected by interannual variations in climate are likely show differences in dispersal patterns.

Considering the 14 outlier loci, the minimum sea surface temperatures recorded in April and in September 2014 explained most of the genetic variance observed within this data set. In particular, the Burry Inlet was strongly associated with the SSTs in September, which was warmer compared with SSTs recorded at other locations for the same time (Figure S1). If cockles in the Burry Inlet are indeed adapted to warmer sea surface temperatures than populations at sites further west or north (and especially compared with SSTs at Gann), it could be speculated that this may explain the maintenance of genetic differentiation between the Burry Inlet and the Gann Estuary despite their spatial proximity and the potential for high connectivity predicted by the model (Figure 3 and Figure S5). Larvae dispersing from Gann to the Burry may not be able to survive postrecruitment given selective pressure against them by higher temperatures. The data collected in this study is not suitable to test this hypothesis, but future investigations of local adaptation of bivalves in the Irish Sea should take these findings into account. Because of the lack of a reference genome and because the sampling locations are not representative of an environmental gradient, our data cannot be used to test this hypothesis of adaptation.

### 4.2 Implications for management

The results from our modelling are likely to be relevant to fishery management in terms of the potential seasonal and interannual variability in larval supply to cockle grounds. This study demonstrates the existence of three distinct units of cockles using both genomic tools and larval dispersal modelling. As with other recent studies (Lal, Southgate, Jerry, Bosserelle, & Zenger, 2017) these findings have important implications for fishery management (Coscia et al., 2012; Miller et al., 2013) and how fisheries management can be reconciled with conservation and other activities.

Given the incidence of recurrent mass mortality events at the Burry Inlet (Callaway et al., 2013), the genetic isolation of this cockle fishery implied by the results of this study should be investigated further. This could be achieved by expanding the sampling coverage of the Burry Inlet to multiple sites and years and assessing its connectivity to other nearby cockle beds that have not been included in this study. Future investigations should be aimed at clarifying the role of adaptive divergence into the fine‐scale population dynamics of the common cockle in this area, to improve management while also assessing the role played by diseases and infections. The results from this study highlight the importance of the use of genomic and hydrodynamic data in assessing population structure and connectivity in an exploited and commercially important marine species and may aid in current and long‐term management regimes of this species (Bernatchez et al., 2017; Lal et al., 2017).

## CONFLICT OF INTEREST

None declared.

## Data Availability

The data sets used in this study are available from the Dryad Digital Repository (https://doi.org/10.5061/dryad.ht76hdrbr).

## References

[eva12932-bib-0001] Alexander, D. H. , & Lange, K. (2011). Enhancement to the Admixture algorithm for individual ancestry information. BMC Bioinformatics, 12, 246. 21682921 10.1186/1471-2105-12-246 PMC3146885

[eva12932-bib-0002] Allendorf, F. W. (2017). Genetics and the conservation of natural populations: Allozymes to genomes. Molecular Ecology, 26(2), 420–430. 10.1111/mec.13948 27933683

[eva12932-bib-0003] Araneda, C. , Larraín, M. A. , Hecht, B. , & Narum, S. (2016). Adaptive genetic variation distinguishes Chilean blue mussels (*Mytilus chilensis*) from different marine environments. Ecology and Evolution, 6(11), 3632–3644. 27195104 10.1002/ece3.2110 PMC4851556

[eva12932-bib-0004] Arnaud‐Haond, S. , Duarte, C. M. , Alberto, F. , & Serrão, E. A. (2007). Standardizing methods to address clonality in population studies. Molecular Ecology, 16(24), 5115–5139. 10.1111/j.1365-294X.2007.03535.x 17944846

[eva12932-bib-0005] Baird, N. A. , Etter, P. D. , Atwood, T. S. , Currey, M. C. , Shiver, A. L. , Lewis, Z. A. , … Johnson, E. A. (2008). Rapid SNP discovery and genetic mapping using sequenced RAD markers. PLoS ONE, 3(10), e3376 10.1371/journal.pone.0003376 18852878 PMC2557064

[eva12932-bib-0006] Barbut, L. , Crego, C. G. , Delerue‐Ricard, S. , Vandamme, S. , Volckaert, F. A. M. , & Lacroix, G. (2019). How larval traits of six flatfish species impact connectivity. Limnology and Oceanography, 64(3), 1150–1171. 10.1002/lno.11104

[eva12932-bib-0007] Beaumont, M. A. , & Balding, D. J. (2004). Identifying adaptive genetic divergence among populations from genome scans. Molecular Ecology, 13(4), 969–980. 10.1111/j.1365-294X.2004.02125.x 15012769

[eva12932-bib-0008] Benestan, L. , Gosselin, T. , Perrier, C. , Sainte‐Marie, B. , Rochette, R. , & Bernatchez, L. (2015). RAD genotyping reveals fine‐scale genetic structuring and provides powerful population assignment in a widely distributed marine species, the American lobster (*Homarus americanus*). Molecular Ecology, 24(13), 3299–3315. 25977167 10.1111/mec.13245

[eva12932-bib-0009] Benestan, L. , Quinn, B. K. , Maaroufi, H. , Laporte, M. , Clark, F. K. , Greenwood, S. J. , … Bernatchez, L. (2016). Seascape genomics provides evidence for thermal adaptation and current‐mediated population structure in American lobster (*Homarus americanus*). Molecular Ecology, 25(20), 5073–5092. 27543860 10.1111/mec.13811

[eva12932-bib-0010] Bernatchez, L. , Wellenreuther, M. , Araneda, C. , Ashton, D. T. , Barth, J. M. I. , Beacham, T. D. , … Withler, R. E. (2017). Harnessing the power of genomics to secure the future of seafood. Trends in Ecology & Evolution, 32(9), 665–680. 10.1016/j.tree.2017.06.010 28818341

[eva12932-bib-0011] Bernatchez, S. , Xuereb, A. , Laporte, M. , Benestan, L. , Steeves, R. , Laflamme, M. , … Bernatchez, L. (2019). Seascape genomics of eastern oyster (*Crassostrea virginica*) along the Atlantic coast of Canada. Evolutionary Applications, 12, 587-609. 30828376 10.1111/eva.12741 PMC6383708

[eva12932-bib-0012] Blanchet, F. G. , Legendre, P. , & Borcard, D. (2008). Modelling directional spatial processes in ecological data. Ecological Modelling, 215(4), 325–336. 10.1016/j.ecolmodel.2008.04.001

[eva12932-bib-0013] Blanchet, F. G. , Legendre, P. , Maranger, R. , Monti, D. , & Pepin, P. (2011). Modelling the effect of directional spatial ecological processes at different scales. Oecologia, 166(2), 357–368. 10.1007/s00442-010-1867-y 21170750

[eva12932-bib-0014] Bode, M. , Leis, J. M. , Mason, L. B. , Williamson, D. H. , Harrison, H. B. , Choukroun, S. , & Jones, G. P. (2019). Successful validation of a larval dispersal model using genetic parentage data. PLOS Biology, 17(7), e3000380 10.1371/journal.pbio.3000380 31299043 PMC6655847

[eva12932-bib-0015] Borcard, D. , Gillet, F. , & Legendre, P. (2011). Numerical ecology with R. New York, NY: Springer.

[eva12932-bib-0016] Botsford, L. W. , White, J. W. , Coffroth, M.‐A. , Paris, C. B. , Planes, S. , Shearer, T. L. , … Jones, G. P. (2009). Connectivity and resilience of coral reef metapopulations in marine protected areas: Matching empirical efforts to predictive needs. Coral Reefs, 28(2), 327–337. 10.1007/s00338-009-0466-z 22833699 PMC3402229

[eva12932-bib-0018] Burgess, S. D. , Bowring, S. , & Shen, S. (2014). High‐precision timeline for Earth’s most severe extinction. Proceedings of the National Academy of Sciences of the United States of America, 111(9), 3316–3321. 10.1073/pnas.1317692111 24516148 PMC3948271

[eva12932-bib-0019] Callaway, R. , Burdon, D. , Deasey, A. , Mazik, K. , & Elliott, M. (2013). The riddle of the sands: How population dynamics explains causes of high bivalve mortality. Journal of Applied Ecology, 50(4), 1050–1059. 10.1111/1365-2664.12114

[eva12932-bib-0020] Chambers, J. M. (2013). Functions and examples for “software for data analysis” (Version 1.0‐6).R package version 1.0-6. https://CRAN.Rproject.org/package=SoDA

[eva12932-bib-0021] Coscia, I. , Robins, P. , Porter, J. , Malham, S. , & Ironside, J. (2012). Modelled larval dispersal and measured gene flow: Seascape genetics of the common cockle *Cerastoderma edule* in the southern Irish Sea. Conservation Genetics, 14, 451 10.1007/s10592-012-0404-4

[eva12932-bib-0022] Coscia, I. , Vogiatzi, E. , Kotoulas, G. , Tsigenopoulos, C. S. , & Mariani, S. (2012). Exploring neutral and adaptive processes in expanding populations of gilthead sea bream, *Sparus aurata* L., in the North‐East Atlantic. Heredity, 108(5), 537–546. 22126850 10.1038/hdy.2011.120 PMC3331784

[eva12932-bib-0024] Cowen, R. , Gawarkiewicz, G. , Pineda, J. , Thorrold, S. , & Werner, F. (2007). Population connectivity in marine systems: An overview. Oceanography, 20, 14–21. 10.5670/oceanog.2007.26

[eva12932-bib-0025] Cowen, R. K. , & Sponaugle, S. (2009). Larval dispersal and marine population connectivity. Annual Review of Marine Science, 1(1), 443–466. 10.1146/annurev.marine.010908.163757 21141044

[eva12932-bib-0026] Dare, P. , Bell, M. C. , Walker, P. H. , & Bannister, R. A. (2004). Historical and current status of cockle and mussel stocks in The Wash. (pp. 85). Lowestoft, UK: CEFAS.

[eva12932-bib-0028] Dray, S. , Bauman, D. , Blanchet, G. , Borcard, D. , Clappe, S. , Guenard, G. , … Wagner, H. H. (2019). adespatial: Multivariate multiscale spatial analysis (Version 0.3‐7). R package version 0.3-7. https://CRAN.R-project.org/package=adespatial

[eva12932-bib-0029] Dray, S. , Legendre, P. , & Peres‐Neto, P. R. (2006). Spatial modelling: A comprehensive framework for principal coordinate analysis of neighbour matrices (PCNM). Ecological Modelling, 196(3), 483–493. 10.1016/j.ecolmodel.2006.02.015

[eva12932-bib-0030] Dray, S. , Pélissier, R. , Couteron, P. , Fortin, M.‐J. , Legendre, P. , Peres‐Neto, P. R. , … Wagner, H. H. (2012). Community ecology in the age of multivariate multiscale spatial analysis. Ecological Monographs, 82(3), 257–275. 10.1890/11-1183.1

[eva12932-bib-0031] Elliott, M. , & Holden, J. (2017). UK sea fisheries statistics 2017 (pp. 174). Newcastle, UK: Marine Management Organisation https://assets.publishing.service.gov.uk/government/uploads/system/uploads/attachment_data/file/742793/UK_Sea_Fisheries_Statistics_2017.pdf

[eva12932-bib-0032] Etter, P. D. , Preston, J. L. , Bassham, S. , Cresko, W. A. , & Johnson, E. A. (2011). Local de novo assembly of RAD paired‐end contigs using short sequencing reads. PLoS ONE, 6(4), e18561 10.1371/journal.pone.0018561 21541009 PMC3076424

[eva12932-bib-0034] Flach, E. C. , & de Bruin, W. (1994). Does the activity of cockles, *Cerastoderma edule* (L.) and lugworms, *Arenicola marina* L., make *Corophium volutator* Pallas more vulnerable to epibenthic predators: A case of interaction modification? Journal of Experimental Marine Biology and Ecology, 182(2), 265–285.

[eva12932-bib-0035] Foll, M. , & Gaggiotti, O. (2008). A genome‐scan method to identify selected loci appropriate for both dominant and codominant markers: A Bayesian perspective. Genetics, 180(2), 977–993. 10.1534/genetics.108.092221 18780740 PMC2567396

[eva12932-bib-0036] Ford, J. R. , Shima, J. S. , & Swearer, S. E. (2016). Interactive effects of shelter and conspecific density shape mortality, growth, and condition in juvenile reef fish. Ecology, 97, 1373–1380. 10.1002/ecy.1436 27459768

[eva12932-bib-0037] Frichot, E. , & Francois, O. (2015). LEA: An R package for landscape and ecological association studies. Methods in Ecology and Evolution, 6, 925–929.

[eva12932-bib-0038] Frichot, E. , Mathieu, F. , Trouillon, T. , Bouchard, G. , & Francois, O. (2014). Fast and efficient estimation of individual ancestry coefficients. Genetics, 196, 973–983. 10.1534/genetics.113.160572 24496008 PMC3982712

[eva12932-bib-0039] Gaffney, P. M. (1994). Heterosis and heterozygote deficiencies in marine bivalves: More light? In Beaumont A.R. (Ed.), Genetics and evolution of aquatic organisms (pp.146-153). London, UK: Chapman & Hall.

[eva12932-bib-0040] Gimenez, L. (2019). Incorporating the geometry of dispersal and migration to understand spatial patterns of species distributions. Ecography, 42(4), 643–657. 10.1111/ecog.03493

[eva12932-bib-0041] Gormley, K. , Mackenzie, C. , Robins, P. , Coscia, I. , Cassidy, A. , James, J. , … Porter, J. (2015). Connectivity and dispersal patterns of protected biogenic reefs: Implications for the conservation of *Modiolus modiolus* (L.) in the Irish Sea. PLoS ONE, 10(12), e0143337 10.1371/journal.pone.0143337 26625263 PMC4666665

[eva12932-bib-0042] Gosselin, T. (2019). assigner: Assignment analysis with GBS/RAD data using R (version 0.5.6). 10.5281/zenodo.592677

[eva12932-bib-0043] Goudet, J. (2005). Hierfstat, a package for r to compute and test hierarchical F‐statistics. Molecular Ecology Notes, 5(1), 184–186. 10.1111/j.1471-8286.2004.00828.x

[eva12932-bib-0044] Goudet, J. , & Jombart, T. (2015). hierfstat: Estimation and tests of hierarchical F‐statistics (version 0.04‐22). https://CRAN.R-project.org/package=hierfstat

[eva12932-bib-0046] Grummer, J. A. , Beheregaray, L. B. , Bernatchez, L. , Hand, B. K. , Luikart, G. , Narum, S. R. , & Taylor, E. B. (2019). Aquatic landscape genomics and environmental effects on genetic variation. Trends in Ecology & Evolution, 34(7), 641–654. 10.1016/j.tree.2019.02.013 30904190

[eva12932-bib-0047] Hartnett, M. , Berry, A. , Tully, O. , & Dabrowski, T. (2007). Investigations into the transport and pathways of scallop larvae—the use of numerical models for managing fish stocks. Journal of Environmental Monitoring, 9(5), 403–410. 10.1039/B617035H 17492084

[eva12932-bib-0048] Hedgecock, D. , & Pudovkin, A. I. (2011). Sweepstakes reproductive success in highly fecund marine fish and shellfish: A review and commentary. Bulletin of Marine Science, 87(4), 971–1002. 10.5343/bms.2010.1051

[eva12932-bib-0049] Hervas, A. , Tully, O. , Hickey, J. , O’Keeffe, E. , & Kelly, E. (2008). Assessment, monitoring and management of the Dundalk Bay and Waterford Estuary Cockle (*Cerastoderma edule*) Fisheries in 2007. Fisheries Resource Series, No. 7 (2008), 38pp

[eva12932-bib-0050] Hickin, V. (2013). Fisheries and nature conservation issues of the Cockle Fishery in the Welsh District of the North Western and North Wales Sea Fisheries Committee. Retrieved from https://naturalresources.wales/media/1678/review-management-cockle-fisheries-wales.pdf

[eva12932-bib-0051] Horsburgh, K. J. , Hill, A. E. , & Brown, J. (1998). A summer jet in the St George’s Channel of the Irish Sea. Estuarine, Coastal and Shelf Science, 47(3), 285–294. 10.1006/ecss.1998.0354

[eva12932-bib-0052] ICES (2018). Interim report of the Working Group on the Application of Genetics in Fisheries and Aquaculture (WGAGFA). Brest, France. ICES CM 2018/ASG:03. 39pp.

[eva12932-bib-0053] Jombart, T. (2008). adegenet: A R package for the multivariate analysis of genetic markers. Bioinformatics, 24(11), 1403–1405. 10.1093/bioinformatics/btn129 18397895

[eva12932-bib-0054] Jombart, T. , & Ahmed, I. (2011). adegenet 1.3‐1: New tools for the analysis of genome‐wide SNP data. Bioinformatics, 27(21), 3070–3071. 21926124 10.1093/bioinformatics/btr521 PMC3198581

[eva12932-bib-0055] Kamvar, Z. N. , Brooks, J. C. , & Grünwald, N. J. (2015). Novel R tools for analysis of genome‐wide population genetic data with emphasis on clonality. Frontiers in Genetics, 6 https://www.frontiersin.org/articles/10.3389/fgene.2015.00208/full 10.3389/fgene.2015.00208 PMC4462096 26113860

[eva12932-bib-0056] Kamvar, Z. N. , Tabima, J. F. , & Grünwald, N. J. (2014). Poppr: An R package for genetic analysis of populations with clonal, partially clonal, and/or sexual reproduction. PeerJ, 2, e281. 24688859 10.7717/peerj.281 PMC3961149

[eva12932-bib-0057] Lal, M. M. , Southgate, P. C. , Jerry, D. R. , Bosserelle, C. , & Zenger, K. R. (2017). Swept away: Ocean currents and seascape features influence genetic structure across the 18,000 Km Indo‐Pacific distribution of a marine invertebrate, the black‐lip pearl oyster *Pinctada margaritifera* . BMC Genomics, 18 10.1186/s12864-016-3410-y PMC5225542 28073363

[eva12932-bib-0058] Legendre, P. , & Gallagher, E. D. (2001). Ecologically meaningful transformations for ordination of species data. Oecologia, 129(2), 271–280. 10.1007/s004420100716 28547606

[eva12932-bib-0059] Lehnert, S. J. , DiBacco, C. , Van Wyngaarden, M. , Jeffery, N. W. , Ben Lowen, J. , Sylvester, E. V. A. , … Bradbury, I. R. (2019). Fine‐scale temperature‐associated genetic structure between inshore and offshore populations of sea scallop (*Placopecten magellanicus*). Heredity, 122(1), 69–80. 10.1038/s41437-018-0087-9 29773897 PMC6288113

[eva12932-bib-0060] Li, H. , Handsaker, B. , Wysoker, A. , Fennell, T. , Ruan, J. , Homer, N. , … Durbin, R. (2009). The Sequence Alignment/Map format and SAMtools. Bioinformatics, 25(16), 2078–2079. 10.1093/bioinformatics/btp352 19505943 PMC2723002

[eva12932-bib-0061] Longshaw, M. , & Malham, S. (2015). A review of the infectious agents, parasites, pathogens and commensals of European cockles (*Cerastoderma edule* and *C. glaucum*). Journal of the Marine Biological Association of the United Kingdom, 93, 227–247.

[eva12932-bib-0062] Luu, K. , Bazin, E. , & Blum, M. G. B. (2017). pcadapt: an R package to perform genome scans for selection based on principal component analysis. Molecular Ecology Resources, 17, 67–77. 10.1111/1755-0998.12592 27601374

[eva12932-bib-0063] Malham, S. , Hutchinson, T. , & Longshaw, M. (2012). A review of the biology of European cockles (Cerastoderma spp.). Journal of the Marine Biological Association of the United Kingdom, 92, 1563–1584.

[eva12932-bib-0064] Manangwa, O. , De Meeus, T. , Grebaut, P. , Segard, A. , Byamungu, M. , & Ravel, S. (2019). Detecting Wahlund effects together with amplification problems: Cryptic species, null alleles and short allele dominance in *Glossina pallidipes* populations from Tanzania. Molecular Ecology Resources, 19, 757–772. 30615304 10.1111/1755-0998.12989

[eva12932-bib-0065] Maroso, F. , Hillen, J. , Pardo, B. G. , Gkagkavouzis, K. , Coscia, I. , Hermida, M. , … Ogden, R. (2018). Performance and precision of double digestion RAD (ddRAD) genotyping in large multiplexed datasets of marine fish species. Marine Genomics, 39, 64–72. 10.1016/j.margen.2018.02.002 29496460

[eva12932-bib-0066] Martínez, L. , Freire, R. , Arias‐Pérez, A. , Mendez, J. , & Insua, A. (2015). Patterns of genetic variation across the distribution range of the cockle *Cerastoderma edule* inferred from microsatellites and mitochondrial DNA. Marine Biology, 162(7), 1393–1406. 10.1007/s00227-015-2676-y

[eva12932-bib-0067] Martínez, L. , Mendez, J. , Insua, A. , Arias‐Pérez, A. , & Freire, R. (2013). Genetic diversity and population differentiation in the cockle *Cerastoderma edule* estimated by microsatellite markers. Helgoland Marine Research, 67, 179–189. 10.1007/s10152-012-0314-3

[eva12932-bib-0068] Masier, S. , & Bronte, D. (2019). Spatial connectedness imposes local‐ and metapopulation‐level selection on life history through feedbacks on demography. Ecology Letters, 23, 242–253. 10.1111/ele.13421 31742858

[eva12932-bib-0069] McKeown, N. J. , Watson, H. V. , Coscia, I. , Wootton, E. , & Ironside, J. E. (2019). Genetic variation in Irish Sea brown crab (*Cancer pagurus* L.): Implications for local and regional management. Journal of the Marine Biological Association of the United Kingdom, 99(4), 879–886.

[eva12932-bib-0070] Miller, A. D. , Versace, V. L. , Matthews, T. G. , Montgomery, S. , & Bowie, K. C. (2013). Ocean currents influence the genetic structure of an intertidal mollusc in southeastern Australia – Implications for predicting the movement of passive dispersers across a marine biogeographic barrier. Ecology and Evolution, 3(5), 1248–1261. 10.1002/ece3.535 23762511 PMC3678479

[eva12932-bib-0071] Monzón‐Argüello, C. , Dell'Amico, F. , Morinière, P. , Marco, A. , López‐Jurado, L. F. , Hays, G. C. , … Lee, P. L. M. (2012). Lost at sea: Genetic, oceanographic and meteorological evidence for storm‐forced dispersal. Journal of the Royal Society Interface, 9(73), 1725–1732. 10.1098/rsif.2011.0788 PMC3385747 22319111

[eva12932-bib-0072] Morgan, E. , O' Riordan, R. M. , & Culloty, S. C. (2013). Climate change impacts on potential recruitment in an ecosystem engineer. Ecology and Evolution, 3(3), 581–594. 10.1002/ece3.419 23532482 PMC3605848

[eva12932-bib-0073] Nielsen, E. E. , Cariani, A. , Aoidh, E. M. , Maes, G. E. , Milano, I. , Ogden, R. , … Carvalho, G. R. (2012). Gene‐associated markers provide tools for tackling illegal fishing and false eco‐certification. Nature Communications, 3, 851 10.1038/ncomms1845 22617291

[eva12932-bib-0074] Nielsen, E. E. , Hemmer‐Hansen, J. , Larsen, P. F. , & Bekkevold, D. (2009). Population genomics of marine fishes: Identifying adaptive variation in space and time. Molecular Ecology, 18, 3128–3150. 19627488 10.1111/j.1365-294X.2009.04272.x

[eva12932-bib-0075] North, E. , Schlag, Z. , Hood, R. , Li, M. , Zhong, L. , Gross, T. , & Kennedy, V. (2008). Vertical swimming behavior influences the dispersal of simulated oyster larvae in a coupled particle‐tracking and hydrodynamic model of Chesapeake Bay. Marine Ecology Progress Series, 359, 99–115. 10.3354/meps07317

[eva12932-bib-0076] Oksanen, F. , Blanchet, F. G. , Friendly, M. , Kindt, R. , Legendre, P. , Mcglinn, D. , … Wagner, H. (2017). vegan: Community Ecology package. R package version 2.4‐4.

[eva12932-bib-0077] Oksanen, J. , Blanchet, G. F. , Friendly, M. , Kindt, R. , Legendre, P. , McGlinn, D. , & Wagner, H. (2019). vegan: Community Ecology Package. R package version 2, 5–6. https://CRAN.R-project.org/package=vegan

[eva12932-bib-0078] Paris, C. B. , Cowen, R. K. , Claro, R. , & Lindeman, K. C. (2005). Larval transport pathways from Cuban snapper (Lutjanidae) spawning aggregations based on biophysical modeling. Marine Ecology‐Progress Series, 296, 93–106. 10.3354/meps296093

[eva12932-bib-0079] Pingree, R. D. , & Griffiths, D. K. (1979). Sand transport paths around the British Isles resulting from M2 and M4 tidal interactions. Journal of the Marine Biological Association of the United Kingdom, 59(2), 497–513.

[eva12932-bib-0080] Pinsky, M. L. , & Palumbi, S. R. (2014). Meta‐analysis reveals lower genetic diversity in overfished populations. Molecular Ecology, 23(1), 29–39. 10.1111/mec.12509 24372754

[eva12932-bib-0081] R Core Team (2019). R: A language and environment for statistical computing. Retrieved from https://www.R-project.org/

[eva12932-bib-0083] Razgour, O. , Forester, B. , Taggart, J. B. , Bekaert, M. , Juste, J. , Ibáñez, C. , … Manel, S. (2019). Considering adaptive genetic variation in climate change vulnerability assessment reduces species range loss projections. Proceedings of the National Academy of Sciences of the United States of America, 116(21), 10418–10423. 10.1073/pnas.1820663116 31061126 PMC6535011

[eva12932-bib-0084] Robins, P. E. , Neill, S. P. , Giménez, L. , Jenkins, S. R. , & Malham, S. K. (2013). Physical and biological controls on larval dispersal and connectivity in a highly energetic shelf sea. Limnology and Oceanography, 58(2), 505–524. 10.4319/lo.2013.58.2.0505

[eva12932-bib-0085] Robins, P. E. , Neill, S. P. , Lewis, M. J. , & Ward, S. L. (2015). Characterising the spatial and temporal variability of the tidal-stream energy resource over the northwest European shelf seas. Applied Energy, 147, 510–522.

[eva12932-bib-0086] Roesti, M. , Salzburger, W. , & Berner, D. (2012). Uninformative polymorphisms bias genome scans for signatures of selection. BMC Evolutionary Biology, 12(1), 94 10.1186/1471-2148-12-94 22726891 PMC3426483

[eva12932-bib-0087] Rowley, A. F. , Cross, M. E. , Culloty, S. C. , Lynch, S. A. , Mackenzie, C. L. , Morgan, E. , … Malham, S. K. (2014). The potential impact of climate change on the infectious diseases of commercially important shellfish populations in the Irish Sea—a review. ICES Journal of Marine Science, 71(4), 741–759. 10.1093/icesjms/fst234

[eva12932-bib-0088] Schwarz, G. (1978). Estimating the dimension of a model. The Annals of Statistics, 6, 461–464.

[eva12932-bib-0089] Selkoe, K. A. , D’Aloia, C. C. , Crandall, E. D. , Iacchei, M. , Liggins, L. , Puritz, J. B. , … Toonen, R. J. (2016). A decade of seascape genetics: Contributions to basic and applied marine connectivity. Marine Ecology Progress Series, 554, 1–19. 10.3354/meps11792

[eva12932-bib-0090] Simpson, J. H. , & Hunter, J. R. (1974). Fronts in the Irish Sea. Nature, 250(5465), 404 10.1038/250404a0

[eva12932-bib-0092] Teacher, A. G. , André, C. , Jonsson, P. R. , & Merilä, J. (2013). Oceanographic connectivity and environmental correlates of genetic structuring in Atlantic herring in the Baltic Sea. Evolutionary Applications, 6(3), 549–567. 10.1111/eva.12042 23745145 PMC3673481

[eva12932-bib-0093] Thieltges, D. W. (2006). Parasite‐induced summer mortality in the cockle *Cerastoderma edule* by the trematode *Gymnophallus choledochus* . Hydrobiologia, 559(1), 455–461. 10.1007/s10750-005-1345-4

[eva12932-bib-0094] Truelove, N. K. , Box, S. J. , Aiken, K. A. , Blythe‐Mallett, A. , Boman, E. M. , Booker, C. J. , … Stoner, A. W. (2017). Isolation by oceanic distance and spatial genetic structure in an overharvested international fishery. Diversity and Distributions, 23(11), 1292–1300. 10.1111/ddi.12626

[eva12932-bib-0095] Van Wyngaarden, M. , Snelgrove, P. V. R. , DiBacco, C. , Hamilton, L. C. , Rodríguez‐Ezpeleta, N. , Zhan, L. , … Bradbury, I. R. (2018). Oceanographic variation influences spatial genomic structure in the sea scallop, *Placopecten * *magellanicus* . Ecology and Evolution, 8(5), 2824–2841. 29531698 10.1002/ece3.3846 PMC5838053

[eva12932-bib-0096] Viricel, A. , & Rosel, P. E. (2014). Hierarchical population structure and habitat differences in a highly mobile marine species: The Atlantic spotted dolphin. Molecular Ecology, 23(20), 5018–5035. 10.1111/mec.12923 25256360

[eva12932-bib-0097] Watson, H. V. , McKeown, N. J. , Coscia, I. , Wootton, E. , & Ironside, J. E. (2016). Population genetic structure of the European lobster (*Homarus gammarus*) in the Irish Sea and implications for the effectiveness of the first British marine protected area. Fisheries Research, 183, 287–293. 10.1016/j.fishres.2016.06.015

[eva12932-bib-0098] Weir, B. S. , & Cockerham, C. C. (1984). Estimating F‐statistics for the analysis of population structure. Evolution, 38(6), 1358–1370. 28563791 10.1111/j.1558-5646.1984.tb05657.x

[eva12932-bib-0099] Wolff, W. J. (2005). The exploitation of living resources in the Dutch Wadden Sea: A historical overview. Helgoland Marine Research, 59(1), 31–38. 10.1007/s10152-004-0204-4

[eva12932-bib-0100] Woodings, L. N. , Murphy, N. P. , Doyle, S. R. , Hall, N. E. , Robinson, A. J. , Liggins, G. W. , … Strugnell, J. M. (2018). Outlier SNPs detect weak regional structure against a background of genetic homogeneity in the Eastern Rock Lobster, *Sagmariasus * *verreauxi* . Marine Biology, 165(12), 185 10.1007/s00227-018-3443-7

[eva12932-bib-0101] Woolmer, A. (2013). Review of national cockle mortality issues and options for fishery management in Kent and Essex IFCA (p. 56). Retrieved from https://www.kentandessex-ifca.gov.uk/wp-content/uploads/2015/07/report-of-EU-and-UK-cockle-mortality.pdf

[eva12932-bib-0102] Xuereb, A. , Benestan, L. , Normandeau, E. , Curtis, J. M. R. , Bernatchez, L. , & Fortin, M. (2018). Asymmetric oceanographic processes mediate connectivity and population genetic structure, as revealed by RADseq, in a highly dispersive marine invertebrate (*Parastichopus californicus*). Molecular Ecology, 27(10), 2347–2364. 29654703 10.1111/mec.14589

[eva12932-bib-0103] Young, E. F. , Belchier, M. , Hauser, L. , Horsburgh, G. J. , Meredith, M. P. , Murphy, E. J. , … Carvalho, G. R. (2015). Oceanography and life history predict contrasting genetic population structure in two Antarctic fish species. Evolutionary Applications, 8(5), 486–509. 10.1111/eva.12259 26029262 PMC4430772

