# Supplementary material for: Fine‐scale seascape genomics of an exploited marine species, the common cockle Cerastoderma edule, using a multimodelling approach
Source: Evol Appl. 2020 Feb 24;13(8):1854–67. doi: 10.1111/eva.12932 (PMC7463313; doi:10.1111/eva.12932)
Supplement: Supplementary file 1 [file EVA-13-1854-s001.docx]

**SUPPLEMENTARY MATERIAL**

**Table S1** Filtering steps. *#individuals*, remaining number of individuals after each filtering step; *#SNPs* remaining number of SNPs

|  | **# individuals** | **# SNPs** |
| --- | --- | --- |
| **Raw dataset** | 191 | 4271 |
| **Missing data** | 159 | 4271 |
| ***F*_IS_** | 138 | 3488 |
| **MAF 1%** | 138 | 1864 |
| **Linkage Disequilibrium** | 138 | 1775 |
| **Hardy-Weinberg eq.** | 138 | 1725 |

**
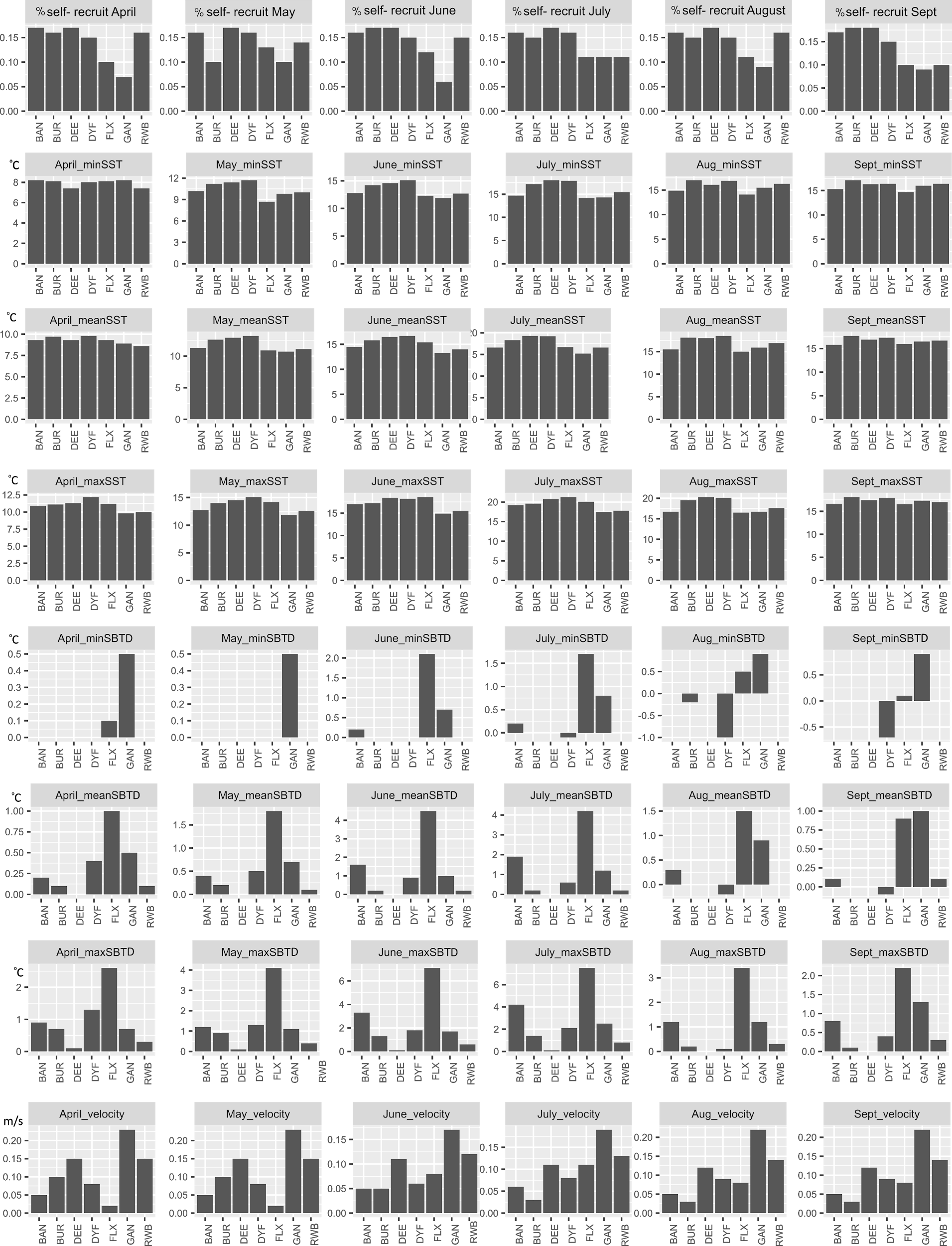
**

***Figure S1****:* Environmental parameters for each location, and each month used in the environmental association analysis.

*
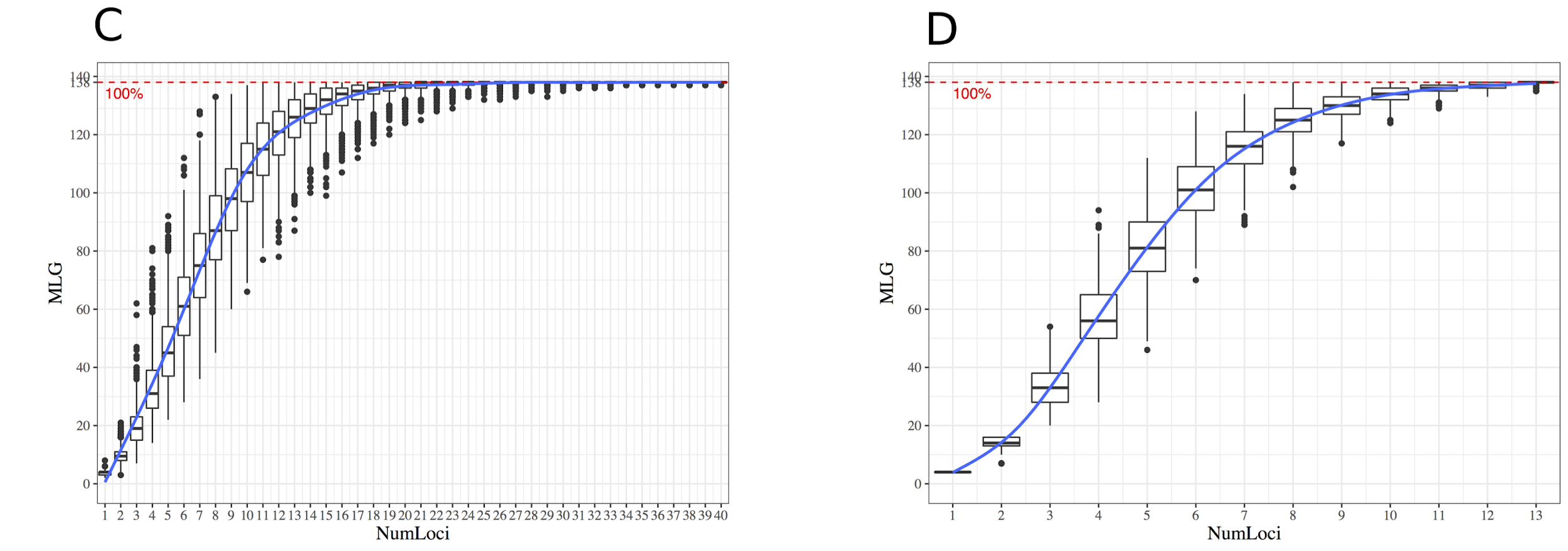
*

***Figure S2:*** *Genotype accumulation curves for neutral (A) and outlier (B) datasets. On the Y axis, the number of multilocus genotypes and on the X axis, the number of loci.*


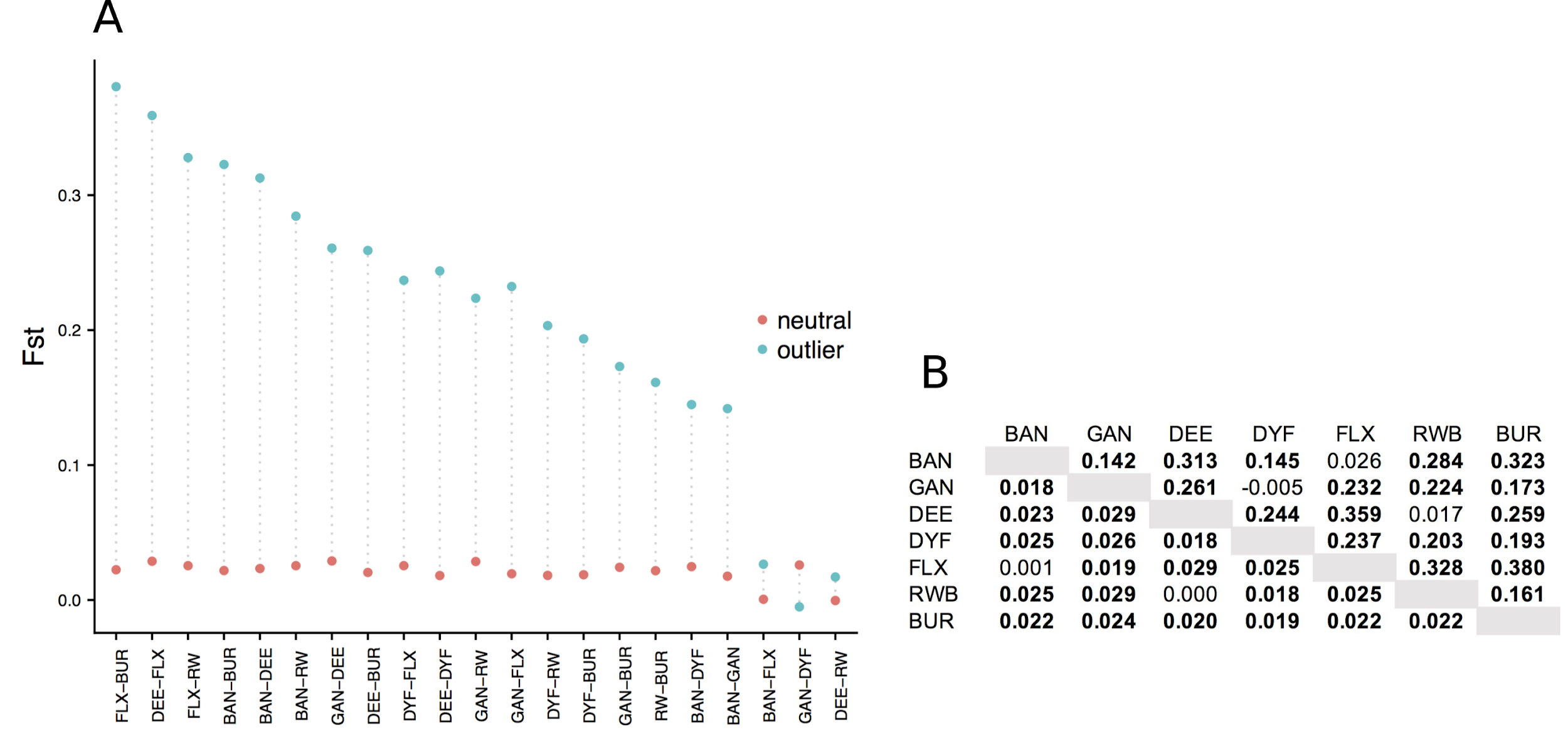


***Figure S3:*** *Pairwise Fst calculated for neutral and outlier datasets. The graph (A) shows which pairs have the highest difference between the two. In the table (B) the relative values for neutral (lower diagonal) and outliers (upper diagonal). In bold, values that are significant (95% confidence interval).*

*
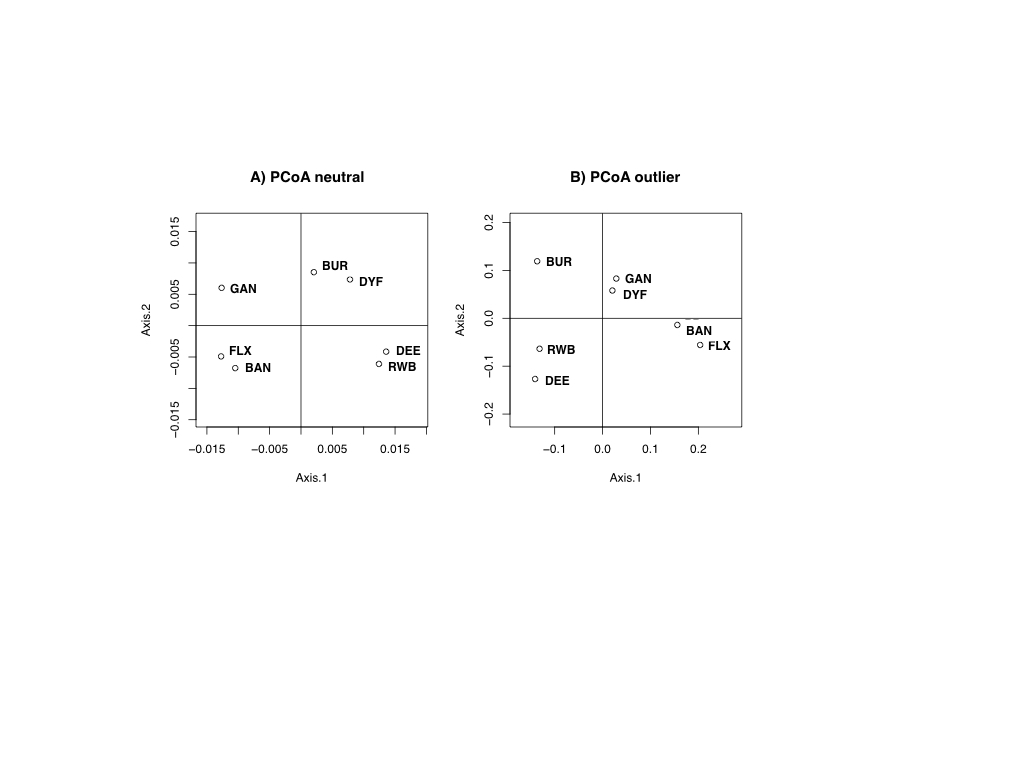
*

***Figure S4:*** Principal Coordinates Analysis (MDS plots) based on *F*_ST_

**Hydrodynamic modelling**

Bathymetry was provided from EMODnet (EMODnet Portal, September 2015 release) and bottom friction was controlled through a log layer with a non-linear drag coefficient set at 0.0025. Ocean boundary conditions were taken from the Global Seasonal Forecast System (GLOSEA), which includes assimilation of both satellite and in-situ observations. Atmospheric forcing was driven by the European Centre for Medium-Range Weather Forecasts (ECMWF) atmospheric reanalysis product, ERA-Interim (Dee et al., 2011), while river inputs were based predominantly on daily climatology of gauge data. See Graham et al. (2018) for further details of the model development and validation.

**Modelling uncertainty**

The ocean model AMM15 was run in free mode without data-assimilation. When these simulations were compared with a simulation with data-assimilation, for a different year to this study, the inherent biases were small and are not expected to change the advection along the tidal mixing fronts and hence larval transport. It is important to ‘trickle-spawn’ larvae each month over a lunar tidal cycle to capture dispersal variability due to the phasing of spawning relative to the tide. Indeed, the simulated variability in dispersal distance, due to the release day (particles were released daily for 16 days), was of the same order as the variability encountered within each daily cohort of 750 particles after 40 days.

The general agreement in population structure between our genomics and modelling analyses could be interpreted as evidence for a larval transport mechanism that is controlled by ocean dispersion rather than swimming behaviour. In effect, this result suggests that the ocean currents are much stronger than larval swimming speeds.

Although inter-annual variability over a 7 year period was simulated, changes in ocean circulation during spawning cycles due to rare and severe storms create additional uncertainty in larval trajectories, potentially leading to the establishment of new communities which have no clear connection at other times.

Despite the relatively fine spatial resolution of our model (1.5 km with 51 vertical layers), some coastal currents will inevitably be poorly resolved, adding a potential source of uncertainty for larval transport in the coastal zone (Dauhajre et al. 2019). Coastal modelling requires an unstructured grid mapped onto a high-resolution coastal bathymetry and including wetting/drying capabilities within the model and also wave-induced currents. This is a considerable task in terms of validation and application to particle tracking, but should be considered for further studies.

*
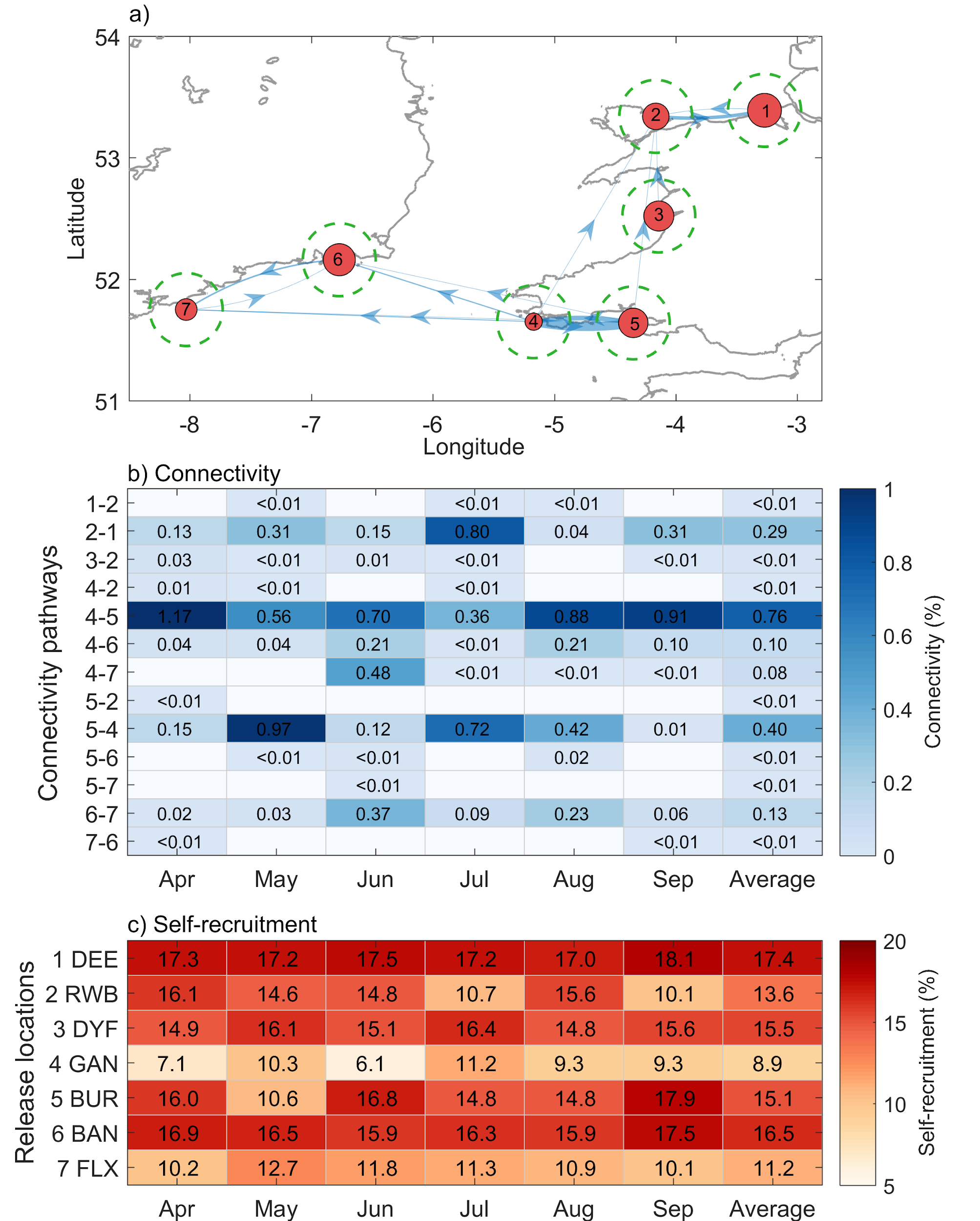
*

***Figure S5:*** *Simulated connectivity between the seven sampled cockle populations. Seasonally-averaged (2014) connectivity networks are shown geographically in (a). The thickness of the pathways in (a) corresponds to the average values in (b). Self-recruitment in (a) is denoted by the size of the red circles which correspond to the average values in (c). The dashed green circles show the settlement radius used to calculate connectivity. Seasonal variability (Apr.-Sep.) is shown for connectivity (b) and self-recruitment (c).*

*
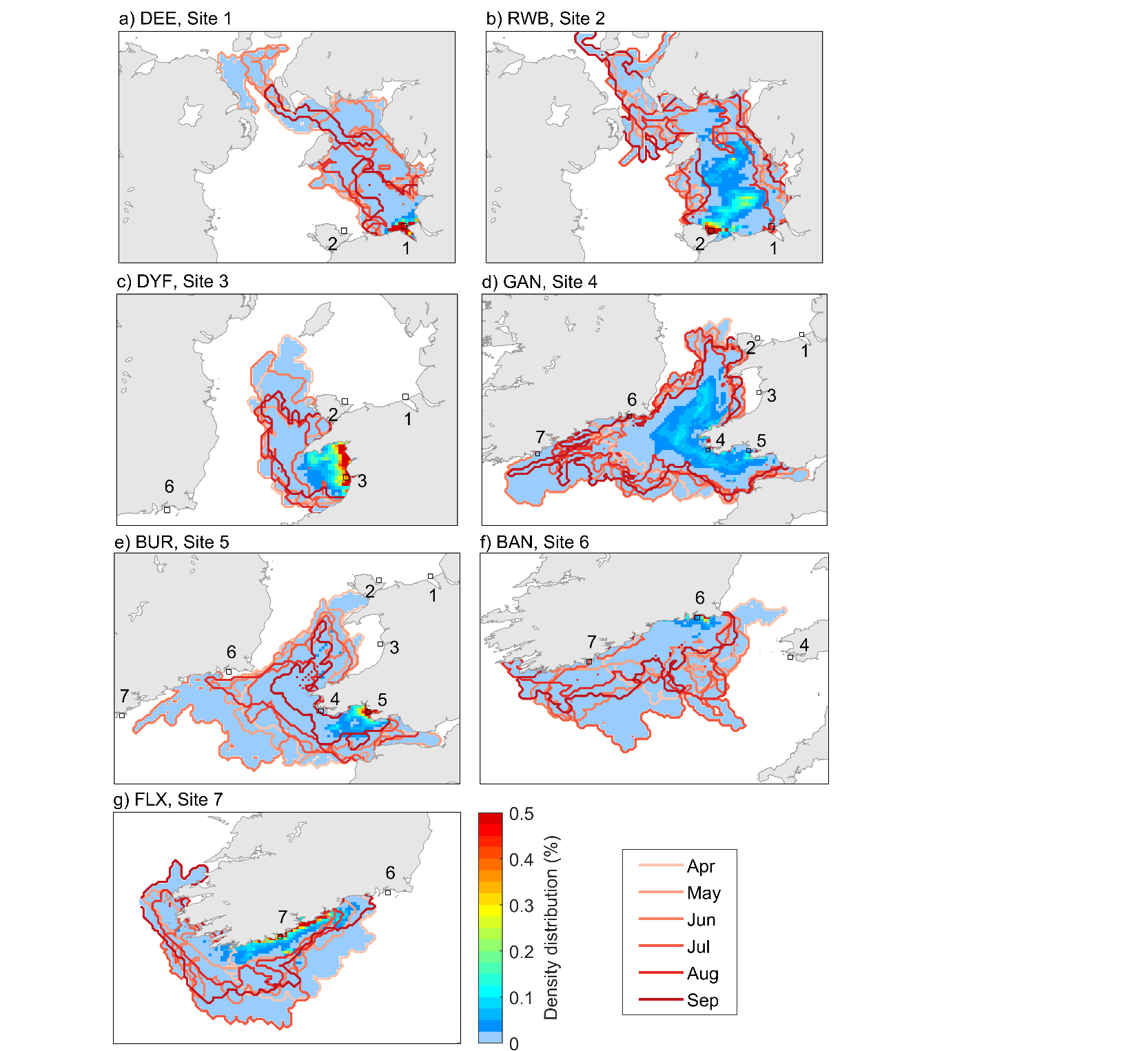
*

***Figure S6:*** *Probability density distribution maps for 2014 showing simulated dispersal probability from release sites 1-7 (black squares). Each panel shows dispersal probability for 11,520,000 particles (12,000 each month × 6 months × 10 settlement days). The superimposed contour lines show the maximum spread per month (April-September), hence an indication of seasonal dispersal variability.*

*
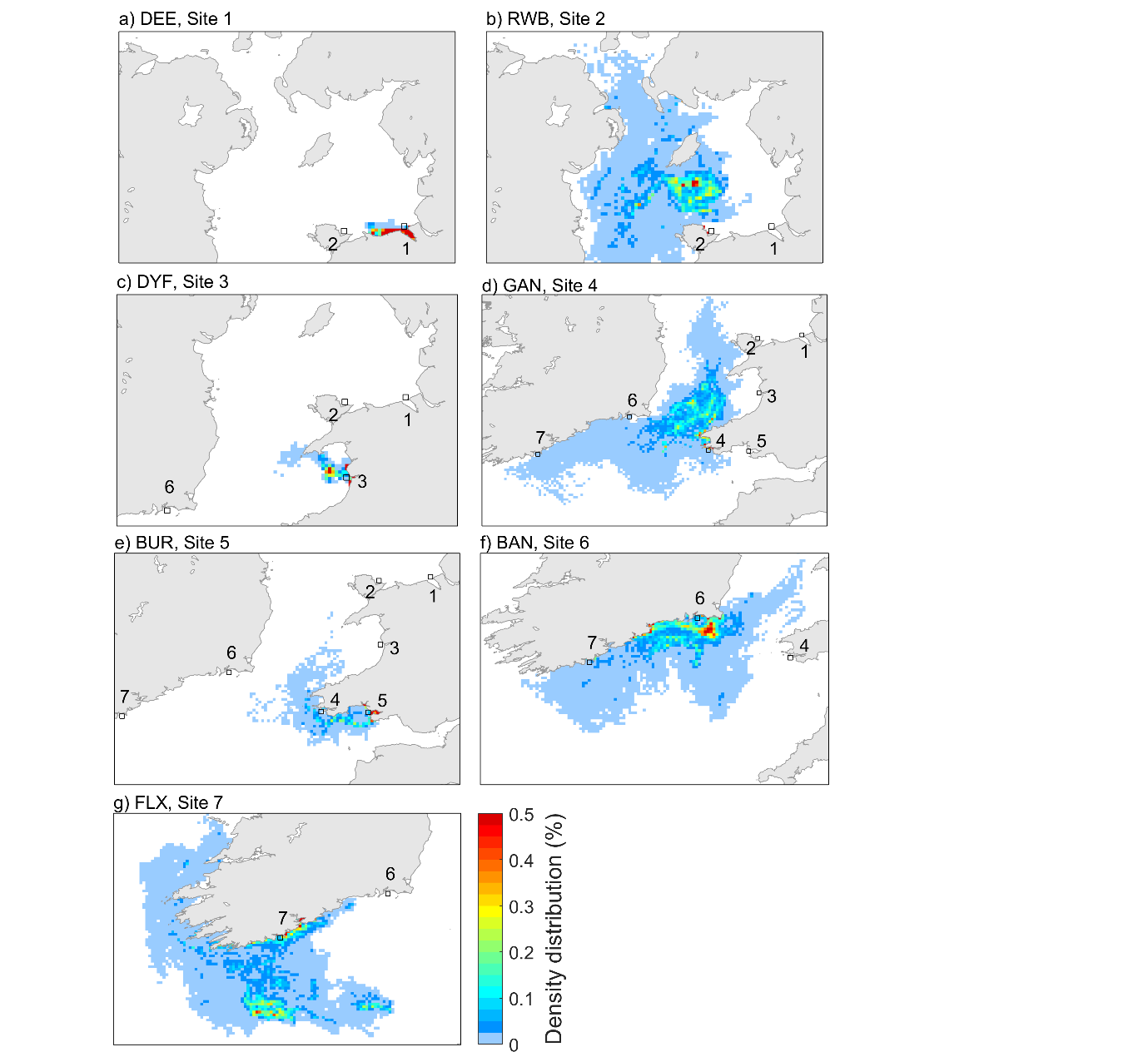

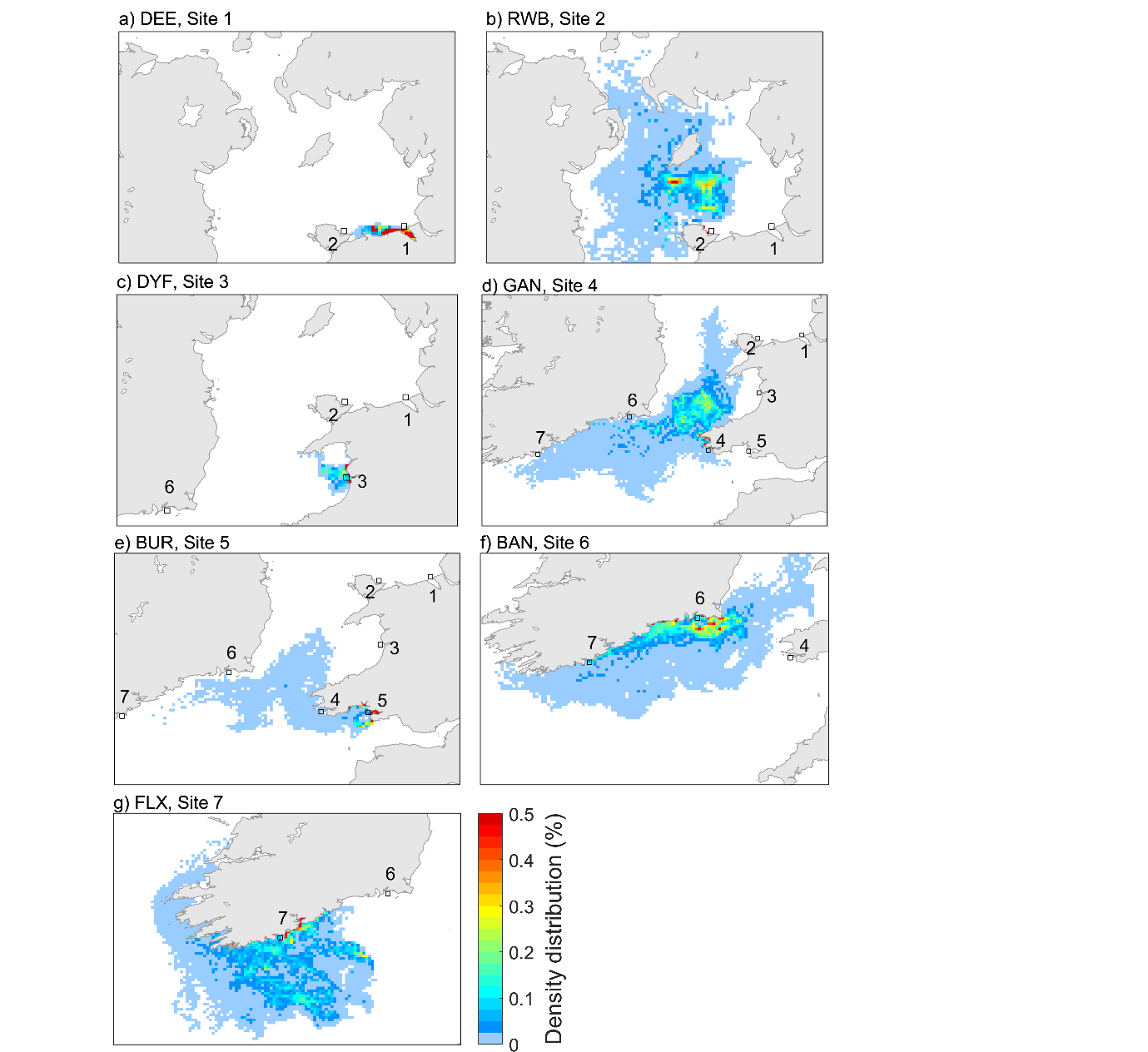

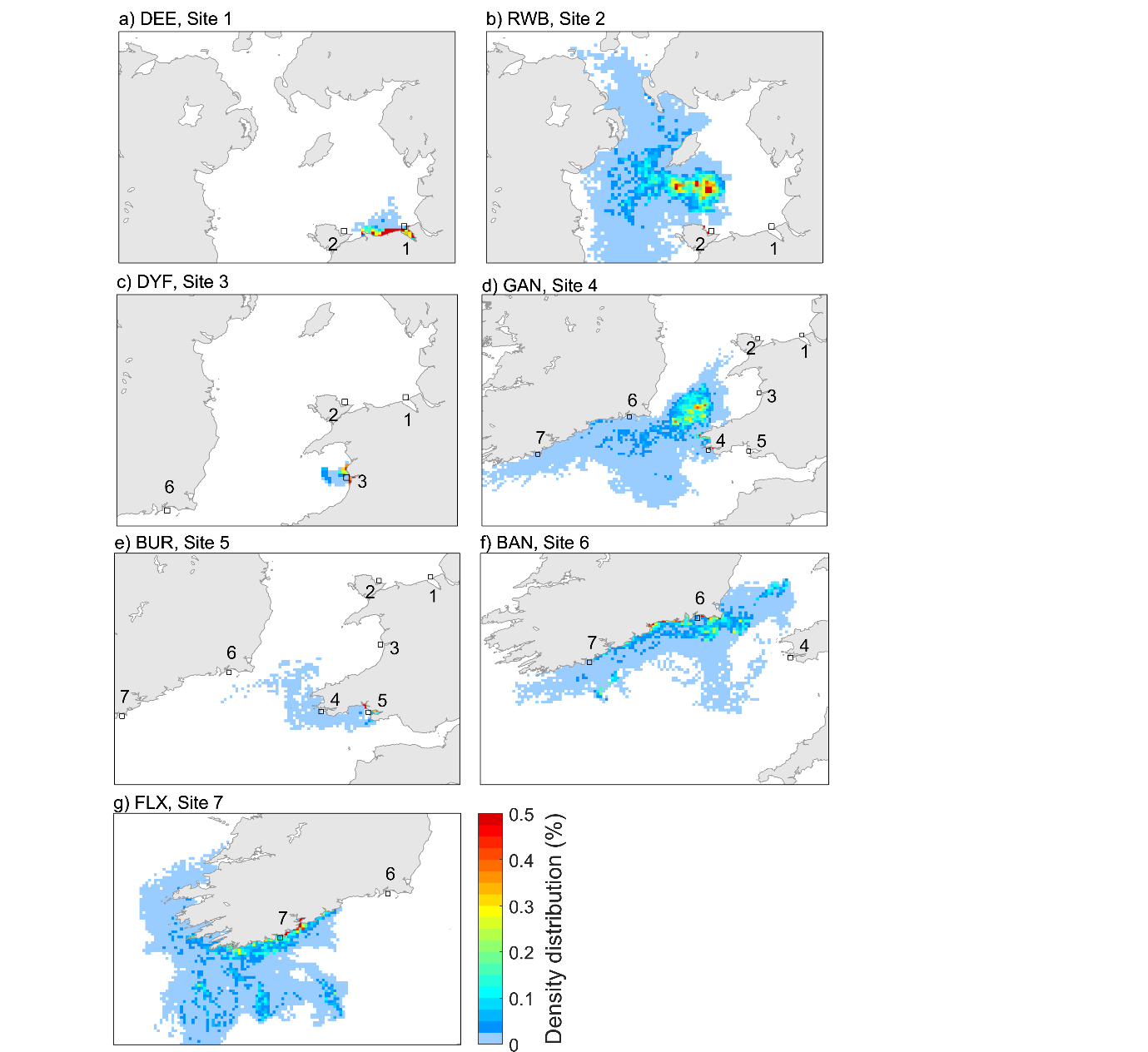

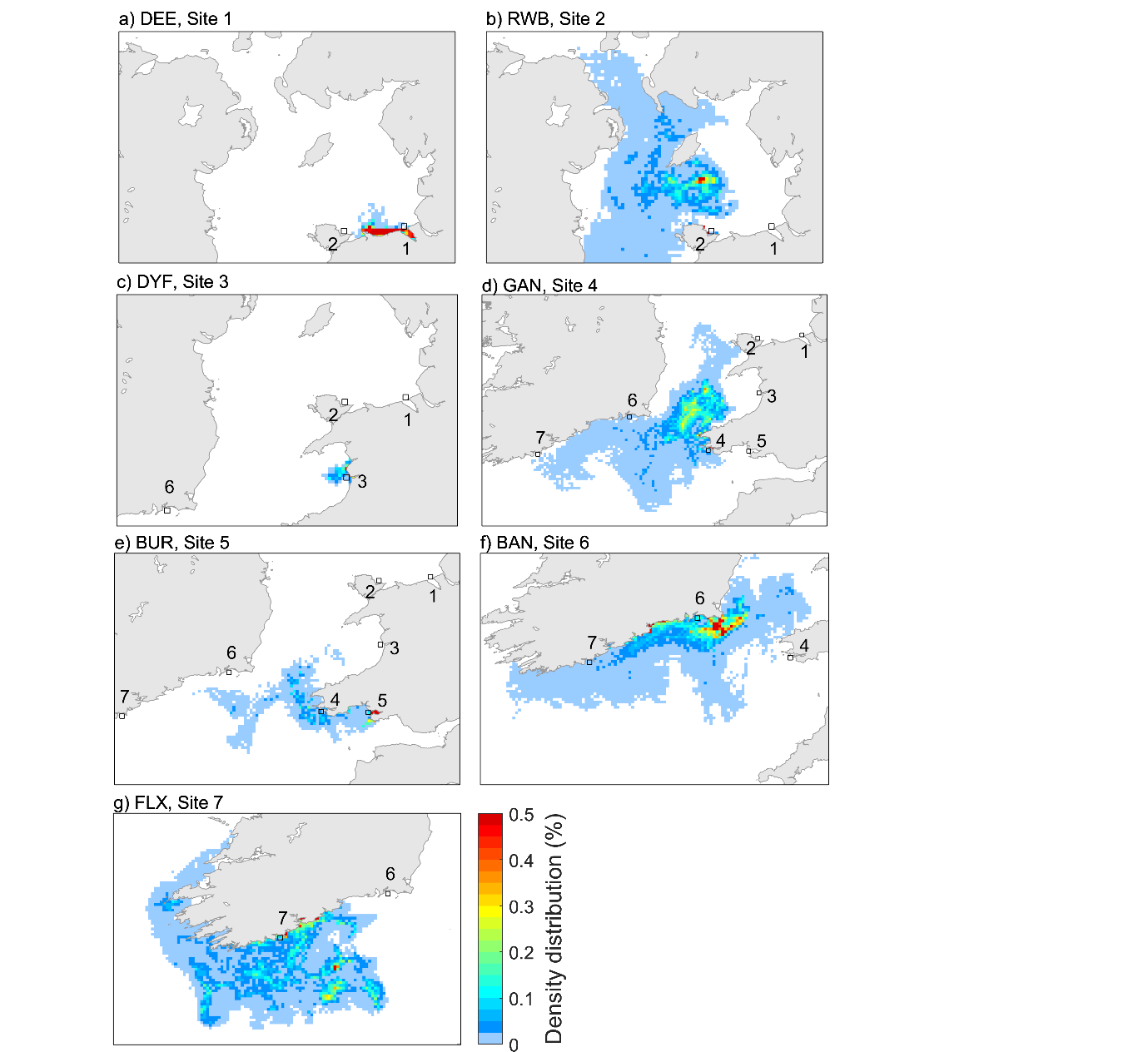

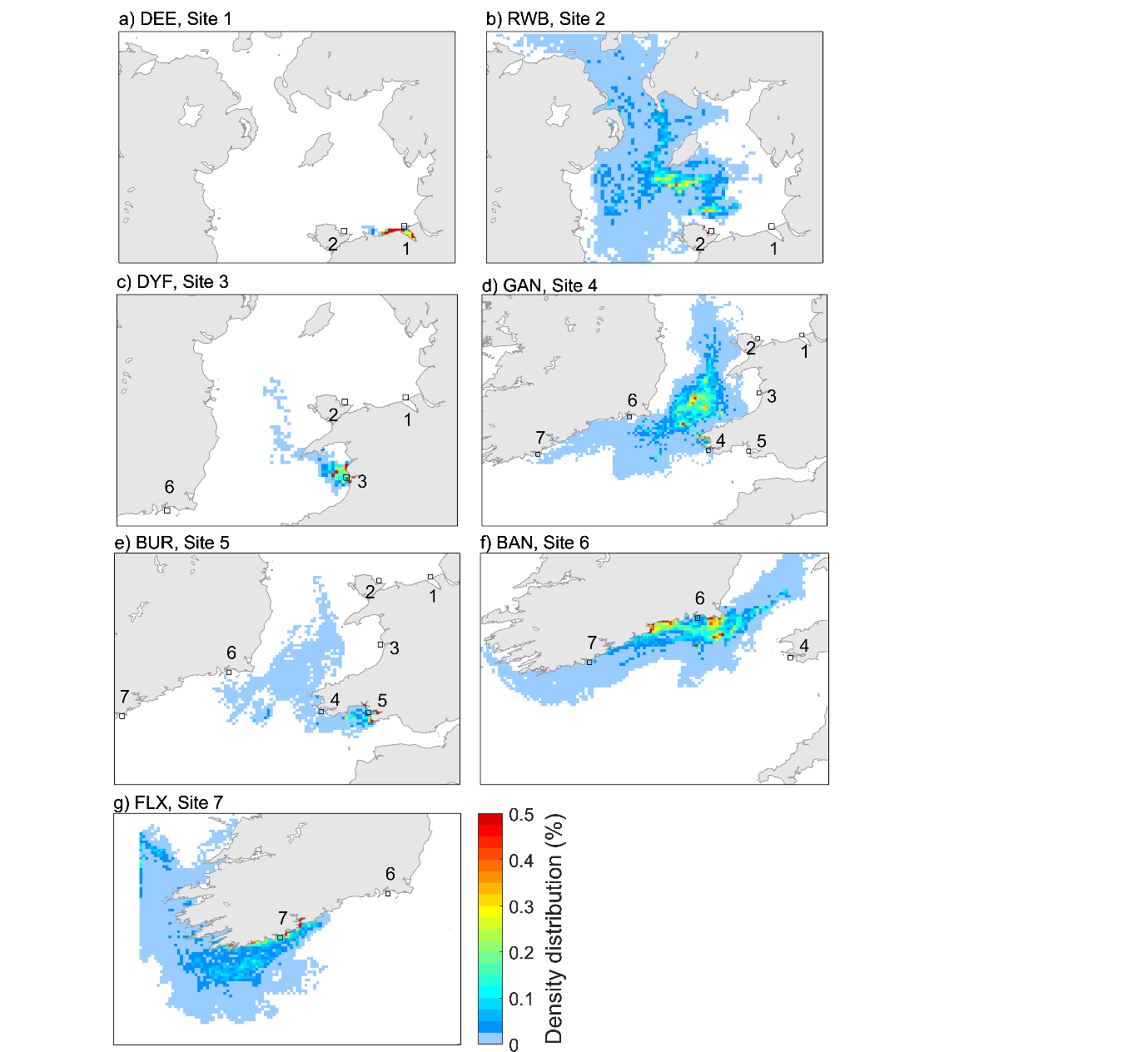

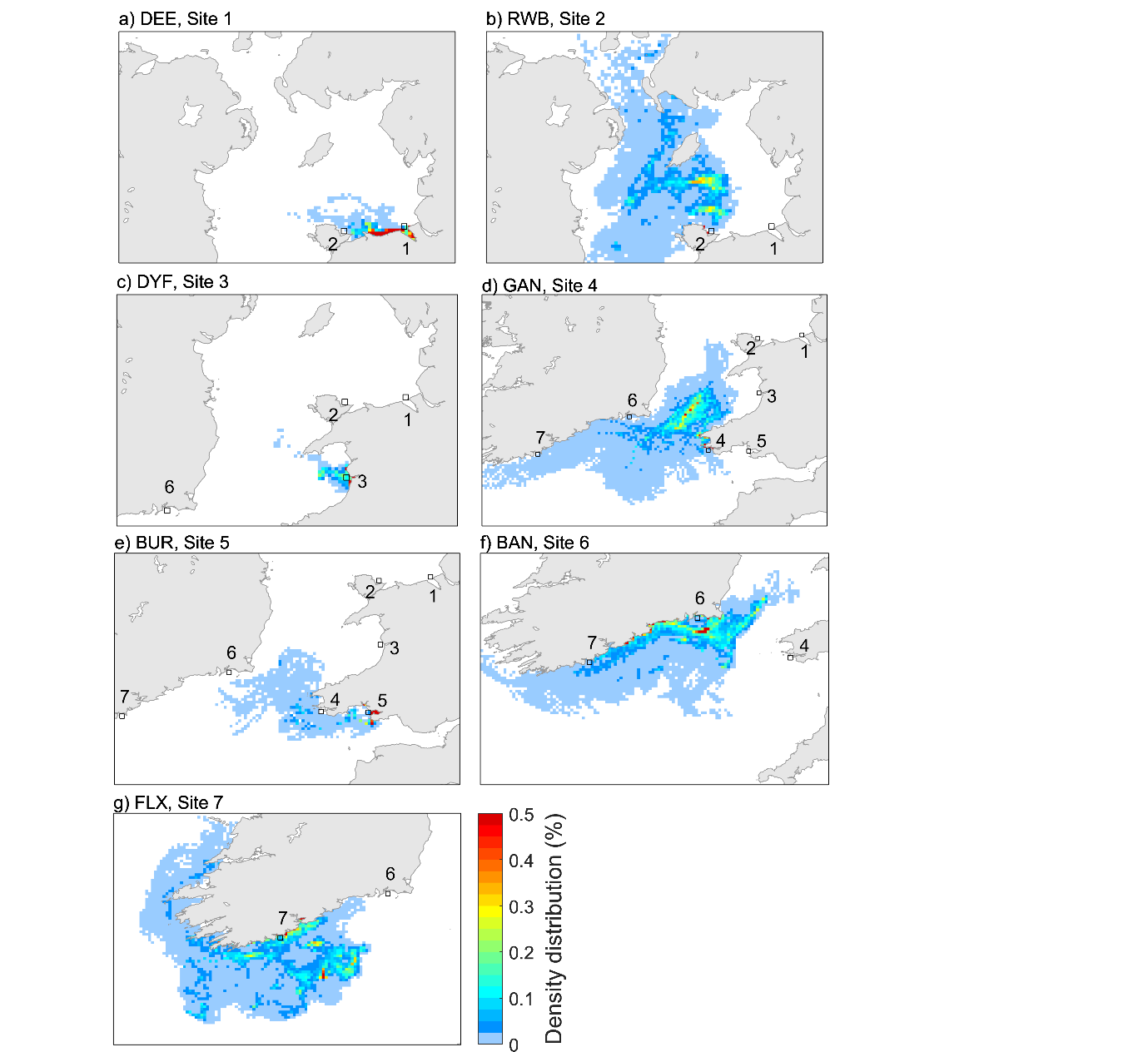

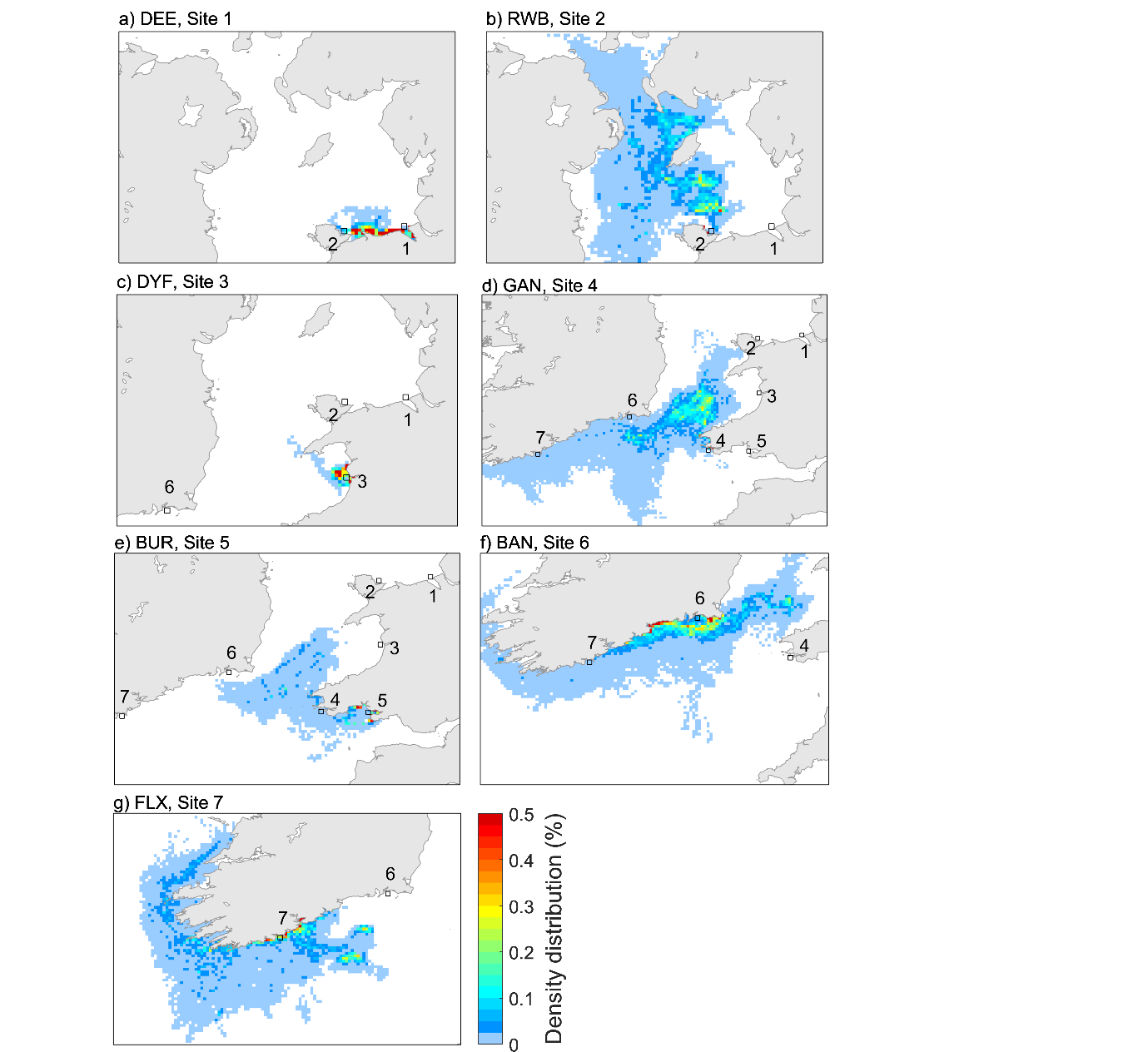
*

***Figure S7.1-S7.7:*** *Probability density distribution maps for 2008-2014 (S7.1-S7.7, respectively) showing simulated dispersal probability from release sites 1-7 (black squares). Each panel shows dispersal probability for 11,520,000 particles (12,000 each month × 6 months × 10 settlement days).*

*
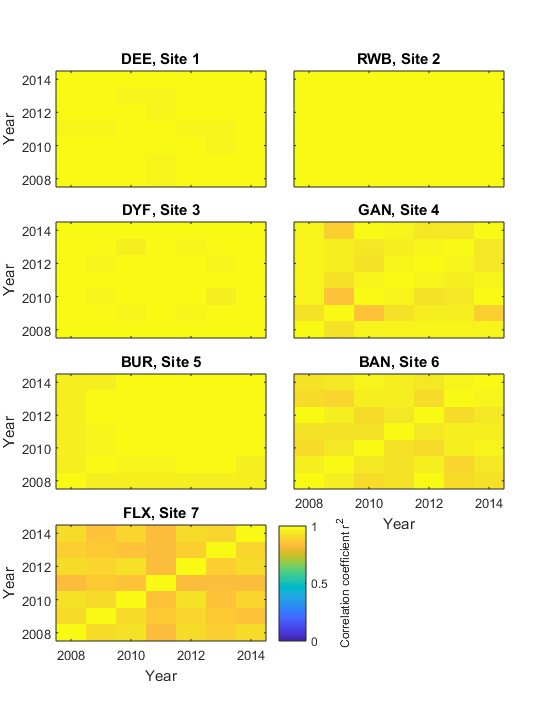
*

***Figure S8:*** *Inter-annual variability in larval dispersal: Correlation coefficients between simulated dispersal maps for daily-averaged current data for years 2008 – 2014. Each panel shows the correlation for the seven release sites during April – September, as described in the Methods.*

*
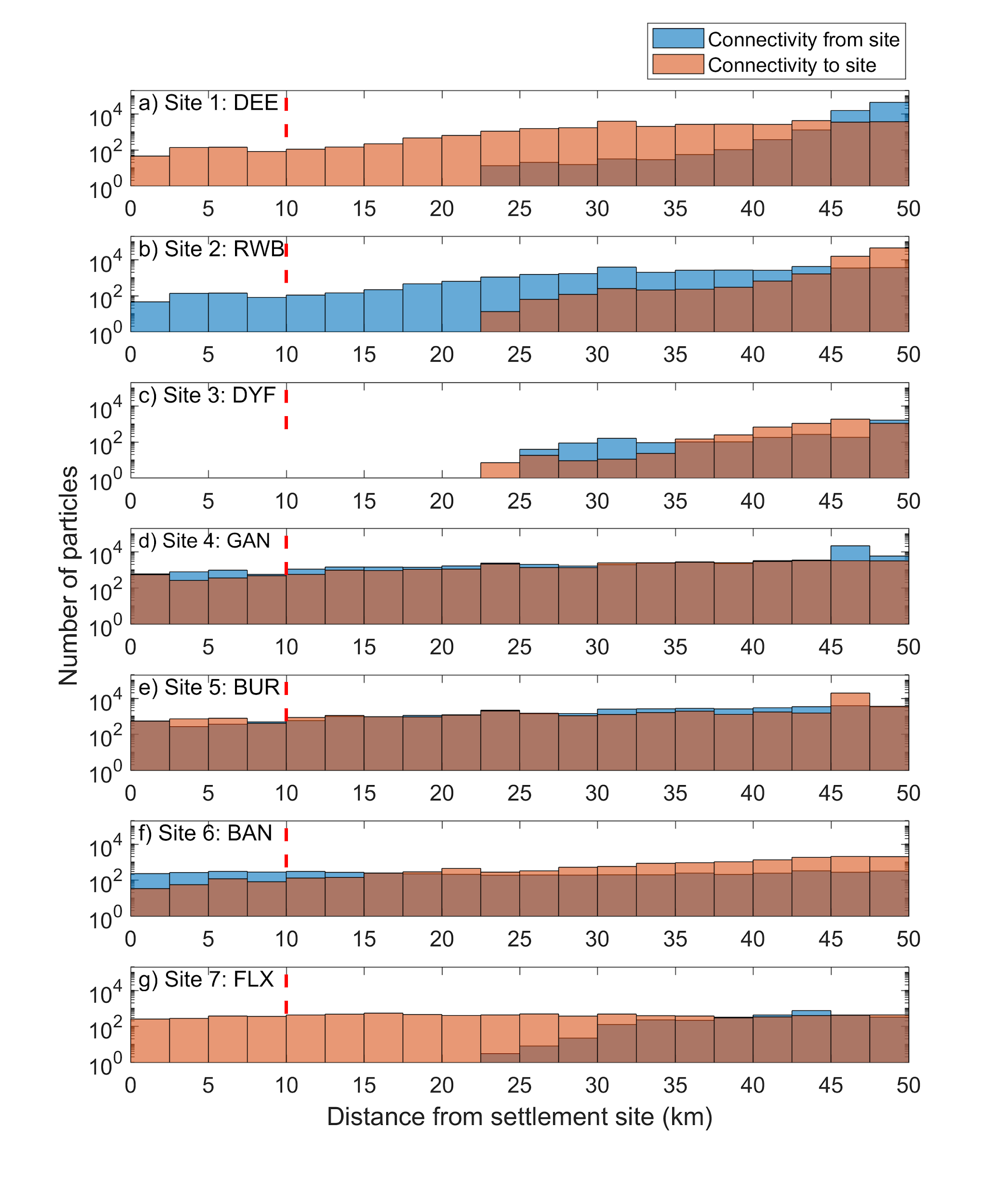
*

***Figure S9****: Histogram plots showing connectivity as a function of distance. For each particle trajectory, the minimum distance to a settlement site during days 30-40 is presented. Blue bars signify the connectivity potential of source sites 1-7 (a-g). Red bars signify the connectivity potential of settlement sites 1-7 (a-g). For example, (a) shows that particles released from DEE simulated no connectivity within 20 km of a settlement site, whereas particles from elsewhere did connect with DEE. The dashed red line denotes our threshold radius for connectivity: 10 km away from each settlement site.*

**References**

Dauhajre, D. P., McWilliams, J. C., & Renault, L. ( 2019). Nearshore Lagrangian connectivity: Submesoscale influence and resolution sensitivity. Journal of Geophysical Research: Oceans, 124, 5180– 5204.
